# **β**-Catenin disruption decreases macrophage exosomal **α**-SNAP and impedes Treg differentiation in acute liver injury

**DOI:** 10.1172/jci.insight.182515

**Published:** 2024-11-19

**Authors:** Ruobin Zong, Yujie Liu, Mengya Zhang, Buwei Liu, Wei Zhang, Hankun Hu, Changyong Li

**Affiliations:** 1 Department of Physiology, Wuhan University TaiKang Medical School (School of Basic Medical Sciences), Wuhan, China.; 2 Department of Pharmacy, Zhongnan Hospital of Wuhan University, Wuhan, China.; 3 Hubei Micro-explore Innovative Pharmaceutical Research Co., Ltd, Wuhan, China.; 4 Suzhou Organ-on-a-Chip System Science and Technology Co., Ltd, Suzhou, China.; 5 Xianning Medical College, Hubei University of Science & Technology, Xianning, China.

**Keywords:** Hepatology, Immunology, Macrophages

## Abstract

Hepatic macrophages and regulatory T cells (Tregs) play an important role in the maintenance of liver immune homeostasis, but the mechanism by which hepatic macrophages regulate Tregs in acute liver injury remains largely unknown. Here, we found that the hepatic Treg proportion and β-catenin expression in hepatic macrophages were associated with acetaminophen- and d-galactosamine/LPS–induced acute liver injury. Interestingly, β-catenin was markedly upregulated only in infiltrating macrophages but not in resident Kupffer cells. Myeloid-specific β-catenin–knockout mice showed an increased inflammatory cell infiltration and hepatocyte apoptosis. Moreover, myeloid β-catenin deficiency decreased the hepatic Treg proportion in the injured liver. Mechanistically, in vitro coculture experiments revealed that macrophage β-catenin modulated its exosome composition and influenced Treg differentiation. Using mass spectrometry–based proteomics, we identified that macrophage β-catenin activation increased the level of exosomal alpha soluble NSF attachment protein (α-SNAP), which in turn promoted Treg differentiation. Overall, our findings demonstrated a molecular mechanism that macrophage β-catenin regulated the Treg proportion in the liver by enhancing the expression of exosomal α-SNAP, providing insights into the pathophysiology of acute liver injury.

## Abstract 2

<div> APAP or D-GalN/LPS induced β-catenin activation in infiltrating macrophages. Furthermore, β-catenin activation regulates the exosomal α-SNAP level of macrophages, thereby influencing Treg differentiation during acute liver injury. </div>

## Graphical abstract

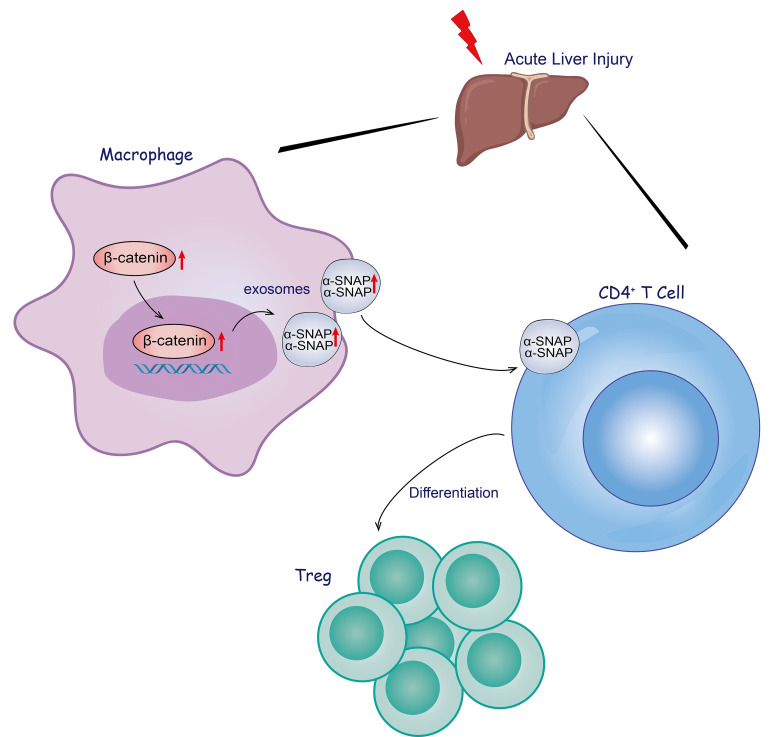

## Introduction

Liver injury and acute liver failure, commonly caused by drug overdose such as acetaminophen (APAP) or by bacterial endotoxin/lipopolysaccharide (LPS), is a significant clinical problem in most countries (1, 2). During the past decades, it has been well documented that liver macrophages play central roles in initiating, perpetuating, and even restricting inflammation in the liver (3). Evidence from mouse models and early clinical studies support the notion that pathogenic macrophage subsets can be potentially translated into novel therapeutic strategies for patients with liver disease (3). However, developing macrophage-targeted therapies against liver diseases faces some challenges. Among them, the main challenge is macrophage heterogeneity and context-dependent functions. Liver macrophages can be divided into resident macrophages (called Kupffer cells) and infiltrating macrophages (called monocyte-derived macrophages), which have highly specialized functions in liver disease (4). Our recent studies also indicated that targeting macrophages could alleviate liver damage during acute liver injury (5–8). However, due to the high plasticity of macrophages in response to their microenvironment, the exact mechanism of macrophage function in various liver diseases is not fully understood. Therefore, further detailed understanding of the distinct pathogenic involvement of liver macrophages may open new perspectives for the treatment of liver diseases.

Regulatory T cells (Tregs), as a distinct lineage of CD4^+^ T lymphocytes, are also essential for the maintenance of immune homeostasis in the liver (9, 10). The primary function of Tregs is immune suppression, by secreting IL-10, TGF-β, and other cytokines. Deficiency or disruption of Tregs was reported to result in autoimmune and inflammatory diseases in humans and other animals (11, 12). In addition, Tregs have been shown to be an important factor in the attenuation of liver injury (13). Indeed, Tregs’ depletion augmented pro-inflammatory cytokines, and exacerbated liver injury, while adoptive transfer of Tregs ameliorated APAP-induced acute liver injury (14).

Although previous reports have shown the regulatory role of liver macrophages on Tregs (15), the mechanism by which macrophages interact with Tregs in acute liver injury has not been fully understood. Exosomes are small, single-membrane, secreted organelles of ~30 to ~200 nm in diameter that have the same topology as the cell and are enriched in selected proteins, lipids, nucleic acids, and glycoconjugates (16). As extracellular carriers, exosomes are important mediators of intercellular communication and play an important role in many aspects of human health and disease, including development, immunity, tissue homeostasis, cancer, and neurodegenerative diseases (16–19). There is growing evidence that macrophage-derived exosomes participate in the regulation of various liver diseases (20, 21). Recently, exosomal long noncoding RNA (lncRNA) H19 has been shown to play a pivotal role in ConA-induced hepatitis through the HIF-1α/p53 signaling pathway (20). In addition, previous evidence suggests that IL-6 could promote palmitic acid induction of miR-223–enriched exosomes in mouse and human macrophages and that its levels correlate positively with miR-223 levels in the serum of patients with metabolic dysfunction–associated steatotic liver disease (21).

The β-catenin signaling is an evolutionary conserved pathway that is necessary for maintaining immune homeostasis (22, 23). As a core component of the cadherin protein complex, the stabilization of β-catenin is essential for the activation of β-catenin signaling (24). Additionally, β-catenin acts as an intracellular signal transducer in the Wnt signaling pathway and is expressed throughout the adult liver (25). Changes in the localization and signaling of β-catenin following liver injury (surgical resection, toxic insult, infection, metabolic insult, or tumor growth) suggest that the β-catenin signaling pathway is an attractive target for liver disease treatment (26, 27). Recently, the β-catenin signaling pathway has been shown to be important in restoring tissue integrity following acute liver toxicity (28), but it remains largely unknown as to how macrophage β-catenin regulates Tregs during acute liver injury.

In this study, we identified a molecular mechanism of cell-cell communication between hepatic macrophages and Tregs during acute liver injury. We demonstrated that the hepatic Treg proportion and β-catenin expression in hepatic macrophages were associated with APAP- and d-galactosamine (D-GalN)/LPS–induced acute liver injury. Importantly, using myeloid-specific β-catenin–knockout (KO) mice and mass spectrometry–based proteomics, we documented that β-catenin modulated macrophage-derived exosomal alpha soluble NSF attachment protein (α-SNAP) level, which in turn influenced Treg differentiation during acute liver injury.

## Results

### β-Catenin is upregulated in infiltrating macrophages but not in resident Kupffer cells during acute liver injury.

**Figure 1:**
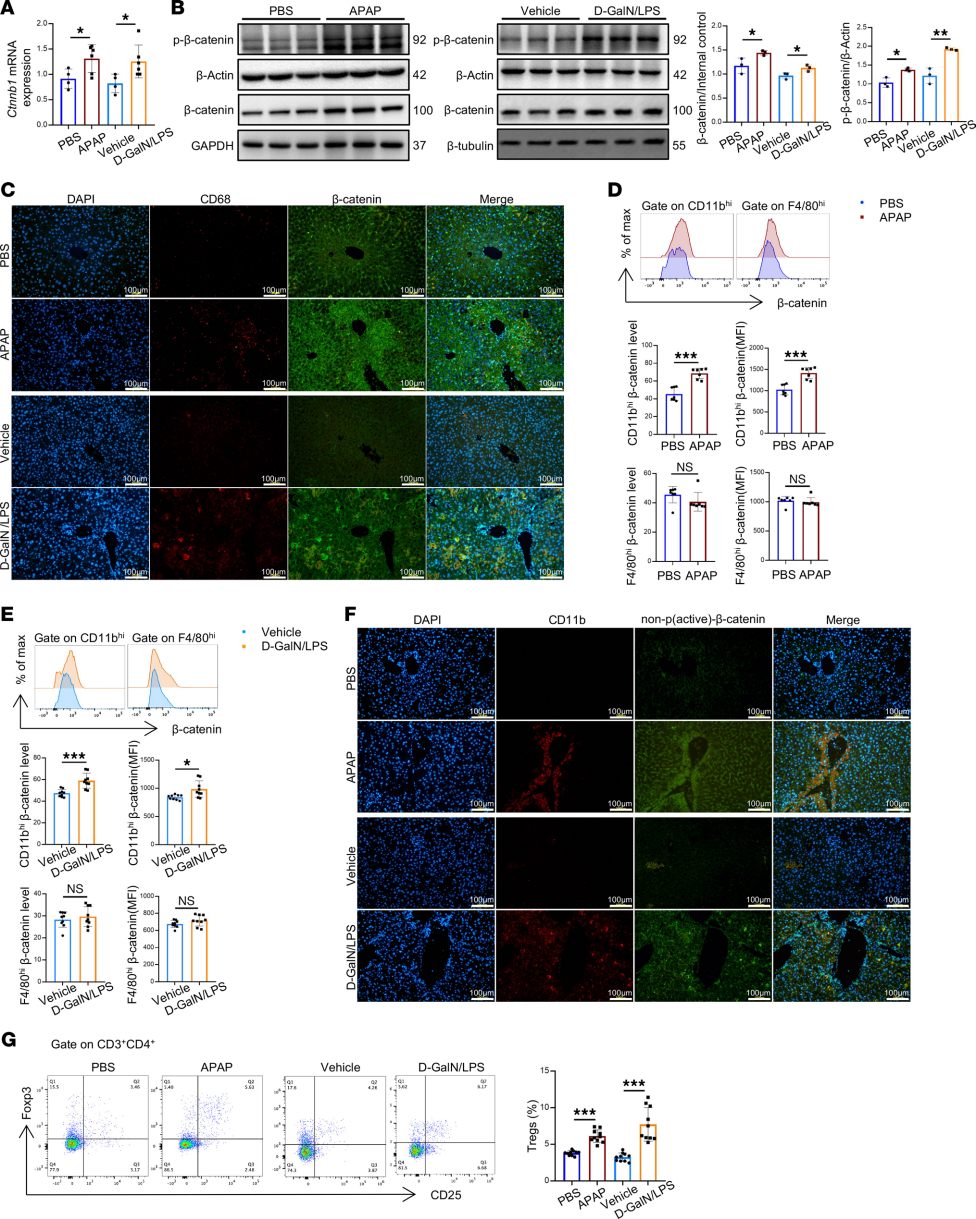
β-Catenin is upregulated in infiltrating macrophages but not in resident Kupffer cells. For APAP overdose–induced acute liver injury, male mice were i.p. injected with APAP (300 mg/kg BW) or the same volume of PBS after overnight fasting, and the mice were sacrificed 24 hours after injection. For D-GalN/LPS–induced acute liver injury, male mice were i.p. injected with 600 mg/kg BW of D-GalN and 30 μg/kg BW of LPS, or the same volume of vehicle, and the mice were sacrificed 5 hours after injection. (**A**) qRT-PCR–assisted detection of β-catenin in the liver tissues (*n* = 4–6 samples/group). qRT, quantitative reverse transcription. (**B**) Western blot analysis of total β-catenin/p–β-catenin (Ser552) expression in the liver tissues. (**C**) Immunofluorescence images of staining with antibodies against CD68 (red) and β-catenin (green). Nuclei were labeled with DAPI (blue). Scale bar, 100 μm. (**D** and **E**) The expression of β-catenin in hepatic macrophages was examined by flow cytometry (*n* = 7–9). (**F**) Immunofluorescence images of staining with antibodies against CD11b (red) and active β-catenin (non-phospho-Ser33/37/Thr41) (green). Nuclei were labeled with DAPI (blue). Scale bar, 100 μm. (**G**) The proportion of hepatic CD25^+^Foxp3^+^ Tregs gated on CD3^+^CD4^+^ T cells was analyzed by flow cytometry (*n* = 10 samples/group). Scale bar, 100 μm. Data are presented as individual values and represent the mean ± SD. *P < 0.05, **P < 0.01, ***P < 0.001 versus nondiabetic controls by 1-way ANOVA in **A**, **B**, and **G** and 2-tailed Student’s *t* test in **D** and **E**.

To investigate the involvement of β-catenin in acute liver injury, we detected the expression of β-catenin in the liver tissues following APAP- and D-GalN/LPS–induced acute liver injury. As shown in Figure 1A, the mRNA level of β-catenin in the liver tissues was markedly increased in injured livers compared with control livers. Meanwhile, we examined the protein levels of β-catenin in liver tissues by Western blot. Clearly, β-catenin was phosphorylated at Ser552 in the liver after APAP or D-GalN/LPS injection, resulting in enhanced activation of hepatic β-catenin (Figure 1B). Using double-immunofluorescence staining, we found that the increased expression of β-catenin was primarily localized in hepatic macrophages (Figure 1C). To explore the expression of β-catenin in different macrophage populations during acute liver injury, we isolated nonparenchymal cells from healthy and injured mouse livers. Interestingly, flow cytometry analysis indicated that β-catenin was markedly upregulated only in infiltrating macrophages (marked by CD11b^hi^F4/80^int^) but not in resident Kupffer cells (marked by CD11b^int^F4/80^hi^) (Figure 1, D and E). Furthermore, we found that the increased expression of active β-catenin (non-phospho-Ser33/37/Thr41) was primarily localized in hepatic CD11b^+^ macrophages (Figure 1F). Consistent with these results, flow cytometry analysis showed that active β-catenin was markedly upregulated in infiltrating macrophages (marked by CD11b^hi^F4/80^int^) (Supplemental Figure 1; supplemental material available online with this article; https://doi.org/10.1172/jci.insight.182515DS1). These observations suggested that β-catenin activation in hepatic infiltrating macrophages is associated with APAP- and D-GalN/LPS–induced liver damage. In addition, flow cytometry analysis revealed that the proportion of CD4^+^CD25^+^Foxp3^+^ Tregs in the liver tissue was markedly increased after APAP or D-GalN/LPS injection (Figure 1G).

### Myeloid β-catenin deficiency aggravates liver injury and reduces hepatic Treg proportion.

**Figure 2:**
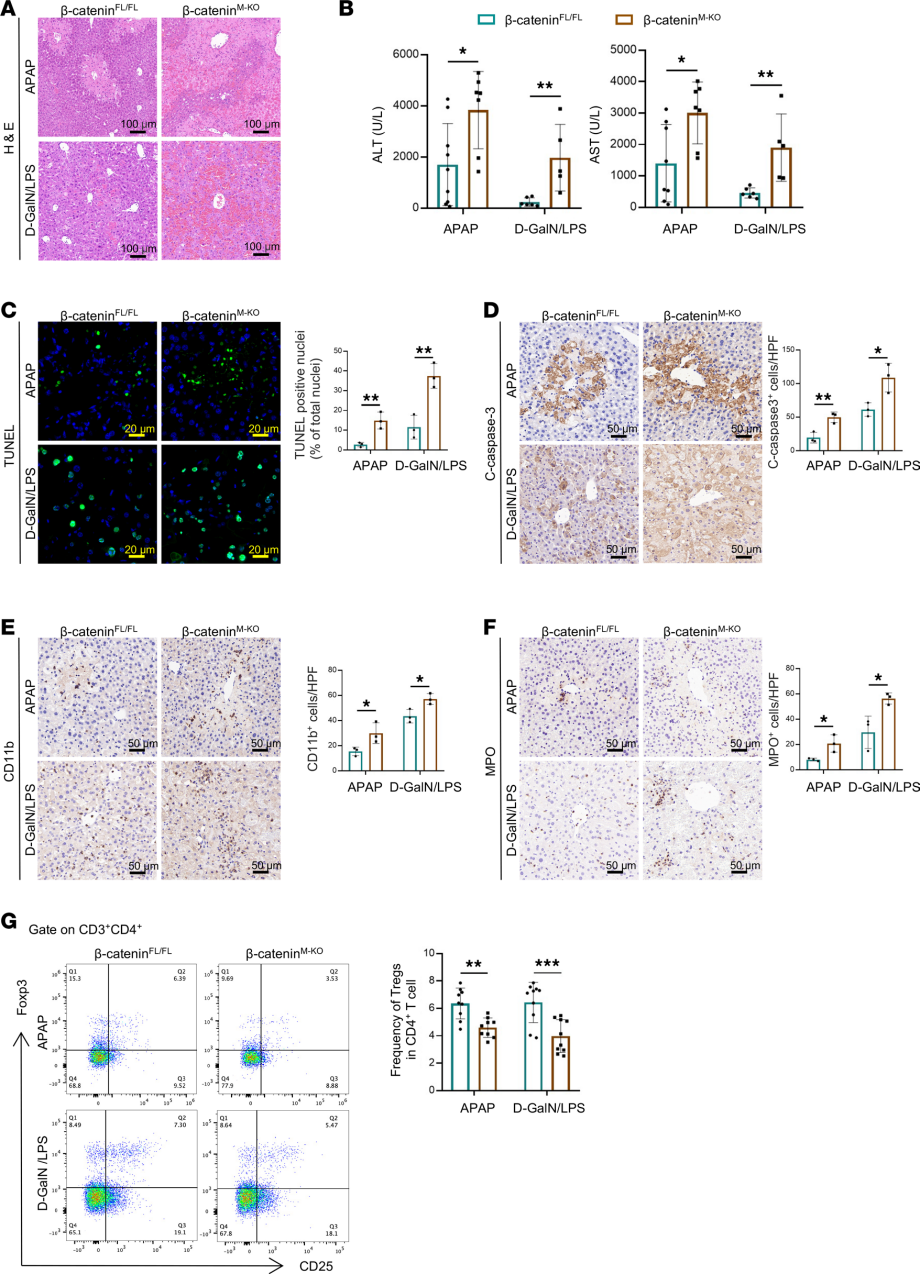
Myeloid β-catenin deficiency aggravates liver injury and reduces hepatic Treg proportion. (**A**) Representative pictures of H&E staining of liver sections. (**B**) Hepatocellular function, as assessed by serum ALT/AST levels (IU/L) (*n* ≥ 5 samples/group). (**C**) Representative images of TUNEL-staining liver sections and quantification of TUNEL-positive nuclei per high-power field for at least 6 fields per sample. (**D**–**F**) Immunohistochemistry staining and quantification of cleaved caspase-3^+^CD11b^+^ macrophages and MPO^+^ neutrophils in the livers. MPO, myeloperoxidase. (**G**) The proportion of hepatic CD25^+^Foxp3^+^ Tregs gated on CD3^+^CD4^+^ T cells was analyzed by flow cytometry (*n* = 9–10 samples/group). Scale bars, 100 μm (**A**); 20 μm (**C**); 50 μm (**D**–**F**). Data are presented as individual values and represent the mean ± SD. *P < 0.05, **P < 0.01, ***P < 0.001 versus nondiabetic controls by 1-way ANOVA.

To further clarify whether β-catenin expression in hepatic macrophages is directly correlated with acute liver injury and the increased Treg proportion, we generated myeloid-specific β-catenin–deficient (β-catenin^M-KO^) and β-catenin–proficient (β-catenin^fl/fl^) mice and subjected them to APAP and D-GalN/LPS toxicity (Supplemental Figure 2). Compared with β-catenin^fl/fl^ mice, the livers in β-catenin^M-KO^ mice showed severe hemorrhage, swollen hepatocytes, larger necrotic area (Figure 2A), and higher serum alanine aminotransferase (ALT) and aspartate aminotransferase (AST) levels (Figure 2B). As apoptosis/necrosis of hepatocytes is the major culprit underlying acute liver injury, we next analyzed the hepatocellular apoptosis by TUNEL staining. Livers in myeloid β-catenin deficiency mice revealed an increased proportion of apoptotic TUNEL^+^ cells compared with control livers (Figure 2C). These data were verified by increased activation of cleaved caspase-3 in β-catenin^M-KO^ compared with that in β-catenin^fl/fl^ mice (Figure 2D). Moreover, the infiltration of CD11b^+^ macrophages and MPO^+^ neutrophils was augmented in β-catenin^M-KO^ mouse liver (Figure 2, E and F). Importantly, flow cytometry analysis showed that myeloid β-catenin disruption markedly reduced Treg proportion in acute liver injury (Figure 2G). These data demonstrated that myeloid β-catenin is essential for controlling the proportion of hepatic Tregs in APAP- and D-GalN/LPS–induced liver injury.

### Macrophage β-catenin disruption depresses Treg differentiation in vitro.

**Figure 3:**
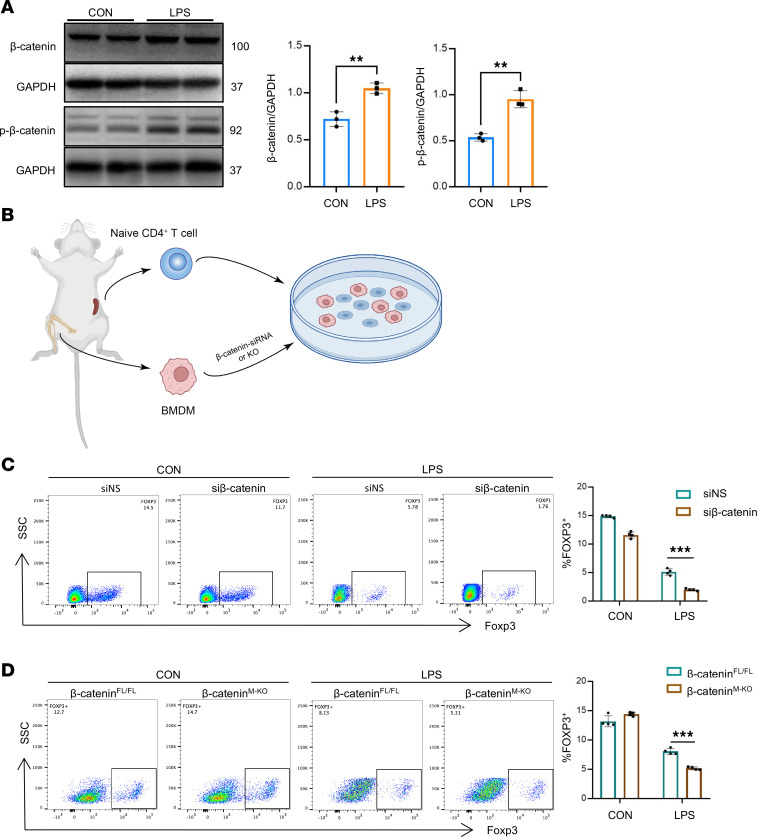
Macrophage β-catenin disruption depresses Treg differentiation in vitro. (**A**) Western blot analysis and quantification of the expression of total β-catenin/p–β-catenin (Ser552) in BMDMs after LPS (500 ng/mL) treatment for 6 hours. (**B**) Coculture diagram. (**C**) The siβ-catenin or siNS-transfected BMDMs were stimulated with LPS (500 ng/mL) or PBS for 6 hours, respectively, then cocultured with naive CD4^+^ T cells for 3 days. The induction of Foxp3^+^ Tregs was analyzed by flow cytometry (*n* = 4). NS, nonsilencing. (**D**) BMDMs from β-catenin^fl/fl^ and β-catenin^M-KO^ mice were stimulated with LPS (500 ng/mL) or PBS for 6 hours, respectively, then cocultured with naive CD4^+^ T cells for 3 days. The induction of Foxp3^+^ Tregs was analyzed by flow cytometry (*n* = 4). Data are presented as individual values and represent the mean ± SD. **P < 0.01, ***P < 0.001 versus nondiabetic controls by 2-tailed Student’s *t* test in **A** and 1-way ANOVA in **C** and **D**.

Next, we further explored the association between macrophage β-catenin activation and Treg differentiation in vitro. Bone marrow–derived macrophages (BMDMs) were isolated from β-catenin^fl/fl^ and β-catenin^M-KO^ mice and stimulated with LPS. Western blot analysis showed that LPS stimulation–induced β-catenin was phosphorylated at Ser552, resulting in augmented β-catenin activation in BMDMs (Figure 3A). To test the hypothesis that macrophage β-catenin regulated Treg differentiation, we established the BMDM/naive CD4^+^ T cell coculture system in vitro (Figure 3B). We disrupted β-catenin in BMDMs using a β-catenin siRNA (Supplemental Figure 3A). Clearly, flow cytometry analysis showed that the Foxp3^+^ Treg population was reduced in response to inhibition of β-catenin in BMDMs (Figure 3C). Consistent with these results, the β-catenin^M-KO^ BMDMs exhibited lower Foxp3^+^ Treg induction and IL-10 levels compared with β-catenin^fl/fl^ BMDMs (Figure 3D and Supplemental Figure 4). Collectively, these data suggested that macrophage β-catenin plays a critical role in Treg differentiation.

### Macrophage β-catenin regulates Treg differentiation via the exosome pathway.

**Figure 4:**
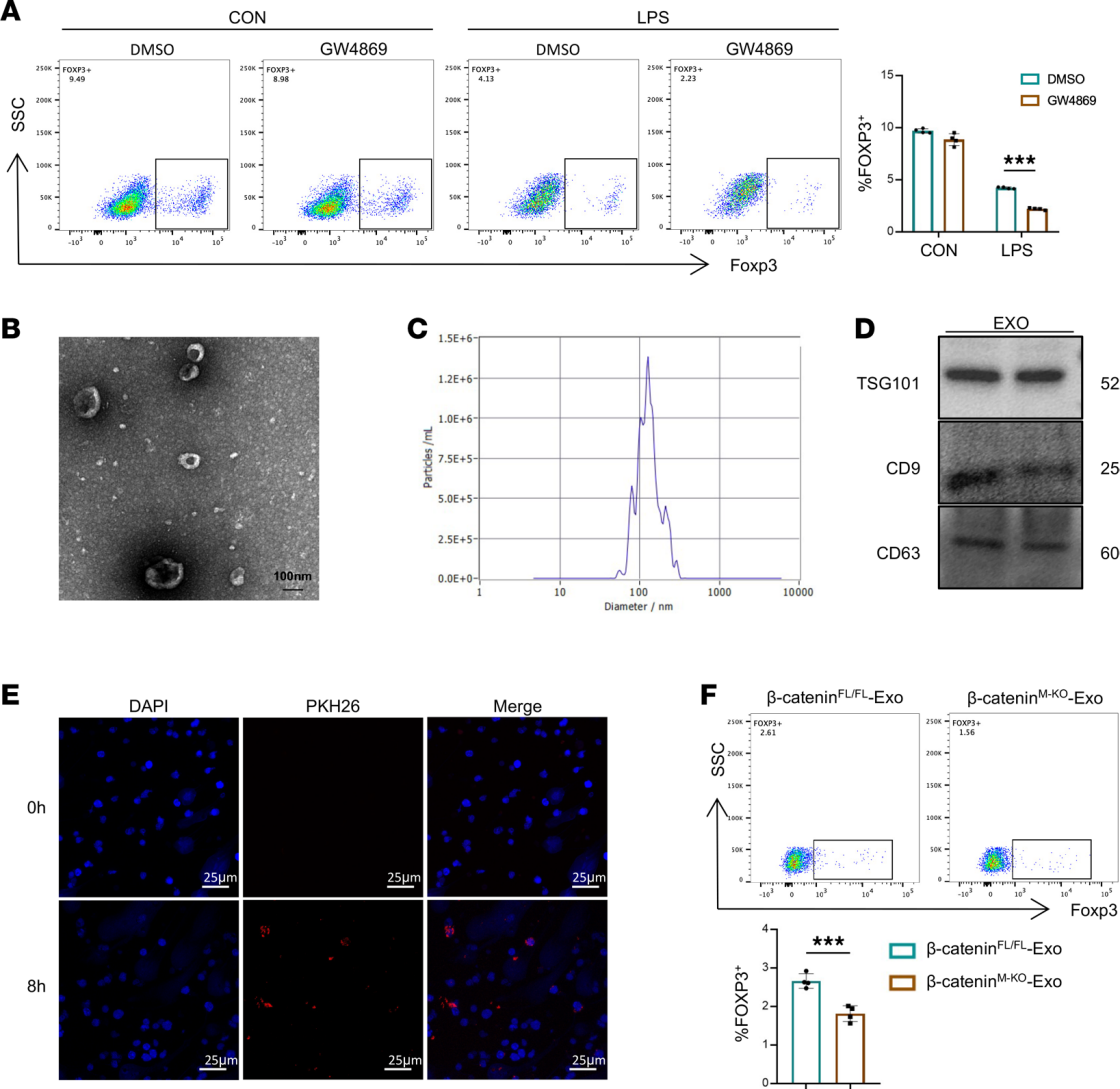
Macrophage β-catenin regulates Treg differentiation via the exosome pathway. (**A**) Naive CD4^+^ T cells were cocultured with BMDMs treated with GW4869 (5 μM) for 3 days, and the induction of Foxp3^+^ Tregs was detected by flow cytometry (*n* = 4). (**B**) Exosomes isolated from BMDM supernatants (BMDM-exosomes) were analyzed by transmission electron microscopy (scale bar, 100 nm). (**C**) Size distribution of exosomes. (**D**) Western blot assay indicated the expression of TSG101, CD9, and CD63 proteins in exosomes. (**E**) BMDM-exosomes were labeled with PKH26 (red) and then cocultured with naive CD4^+^ T cells for 8 hours. The T cells were collected for fluorescence confocal microscopy to detect exosome uptake, and the nuclear location was determined by DAPI (blue) staining (scale bar, 25 μm). (**F**) BMDMs from β-catenin^fl/fl^ and β-catenin^M-KO^ mice were stimulated with LPS (500 ng/mL) or PBS for 6 hours. Then the naive CD4^+^ T cells were cocultured with BMDM exosomes for 3 days. The induction of Foxp3^+^ Tregs was analyzed by flow cytometry (*n* = 4). Data are presented as individual values and represent the mean ± SD. ***P < 0.001 versus nondiabetic controls by 1-way ANOVA in **A** and 2-tailed Student’s *t* test in **F**. Representative of 3 experiments.

Given the potent regulation of macrophage β-catenin on Treg differentiation and that the exosome plays a key role in cell-cell communication and maintenance of liver homeostasis (29, 30), we proposed that macrophage β-catenin may regulate Treg differentiation via the exosome pathway. GW4869 is a commonly used chemical inhibitor that blocks the release of exosomes. As shown in Figure 4A, GW4869 treatment in BMDMs decreased Treg proportion. Next, we isolated and characterized BMDM-derived exosomes. The representative morphology of purified exosomes, a characteristic saucer-like shape, was observed by transmission electron microscopy (Figure 4B). In addition, nanoparticle tracking analysis (NTA) showed that the exosomes had an average diameter ranging from 50 to 150 nm (Figure 4C). Western blotting verified that exosomes expressed and enriched the known exosome markers TSG101, CD9, and CD63 (Figure 4D). To determine whether CD4^+^ T cells can take up BMDM-secreted exosomes, we used red fluorescent dye PKH26 to label the BMDM-derived exosomes and then added them into the culture medium of naive CD4^+^ T cells. Confocal microscopy analysis showed that BMDM-derived exosomes were taken up by naive CD4^+^ T cells after 8 hours of coculture (Figure 4E and Supplemental Figure 5). Furthermore, to determine whether macrophage β-catenin influenced exosome-mediated Treg differentiation, we incubated naive CD4^+^ T cells with exosomes isolated from β-catenin^M-KO^ and β-catenin^fl/fl^ BMDMs. As expected, flow cytometry analysis revealed that β-catenin^M-KO^ BMDM exosomes exhibited lower induction of Foxp3^+^ Tregs compared with β-catenin^fl/fl^ BMDM exosomes (Figure 4F). Taken together, these results suggested that macrophage β-catenin modulates its exosomes and influences Treg differentiation.

### Macrophage β-catenin deletion decreases exosomal α-SNAP and impedes Treg differentiation.

**Figure 5:**
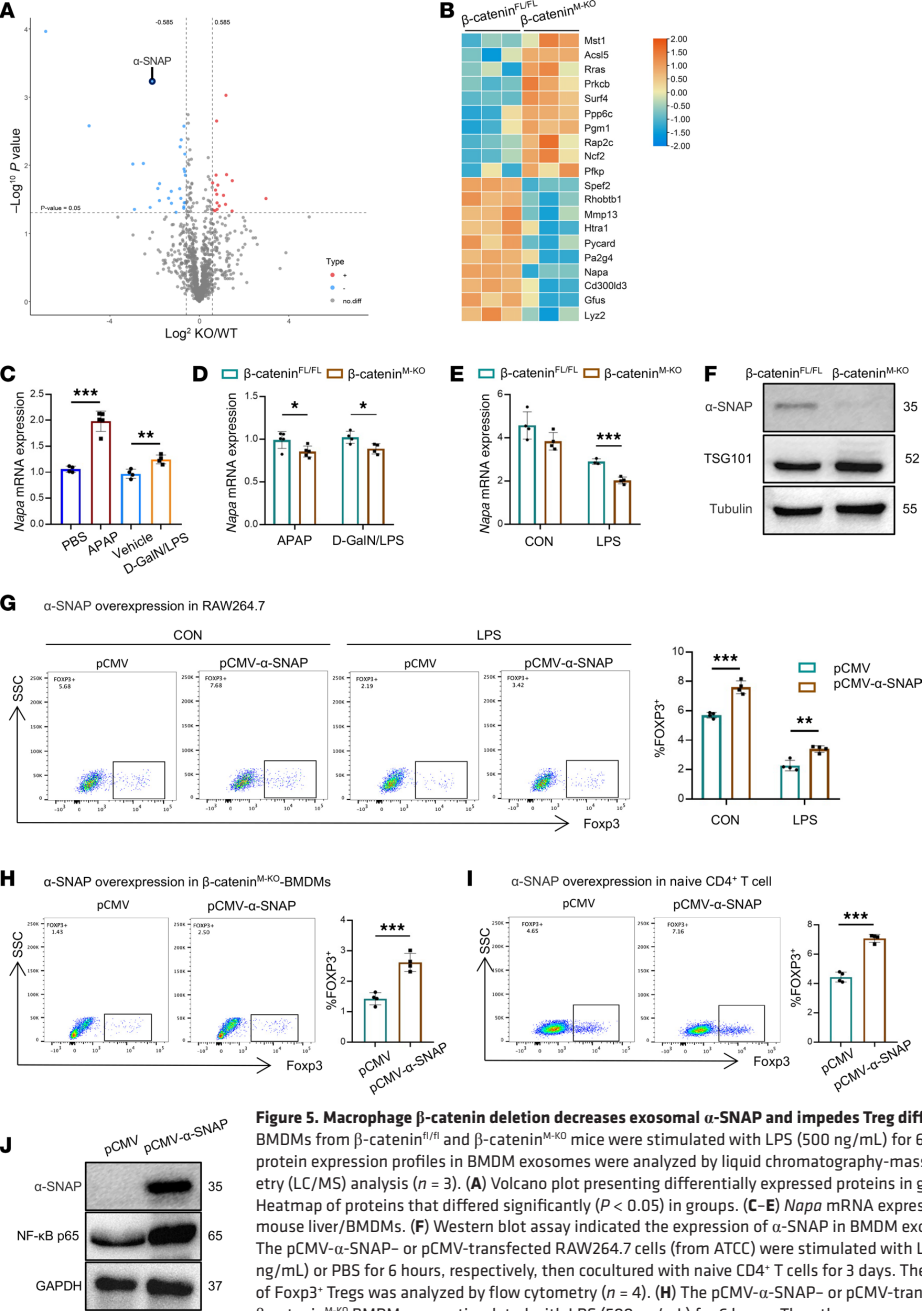
Macrophage β-catenin deletion decreases exosomal α-SNAP and impedes Treg differentiation. BMDMs from β-catenin^fl/fl^ and β-catenin^M-KO^ mice were stimulated with LPS (500 ng/mL) for 6 hours. The protein expression profiles in BMDM exosomes were analyzed by liquid chromatography-mass spectrometry (LC/MS) analysis (*n* = 3). (**A**) Volcano plot presenting differentially expressed proteins in groups. (**B**) Heatmap of proteins that differed significantly (*P* < 0.05) in groups. (**C**–**E**) *Napa* mRNA expression in the mouse liver/BMDMs. (**F**) Western blot assay indicated the expression of α-SNAP in BMDM exosomes. (**G**) The pCMV-α-SNAP– or pCMV-transfected RAW264.7 cells (from ATCC) were stimulated with LPS (500 ng/mL) or PBS for 6 hours, respectively, then cocultured with naive CD4^+^ T cells for 3 days. The induction of Foxp3^+^ Tregs was analyzed by flow cytometry (*n* = 4). (**H**) The pCMV-α-SNAP– or pCMV-transfected β-catenin^M-KO^ BMDMs were stimulated with LPS (500 ng/mL) for 6 hours. Then the exosomes from β-catenin^M-KO^ BMDMs+pCMV and β-catenin^M-KO^ BMDMs+pCMV-α-SNAP were collected to coculture with naive CD4^+^ T cells for 3 days. The induction of Foxp3^+^ Tregs was analyzed by flow cytometry (*n* = 4). (**I**) The induction of Foxp3^+^ Tregs was detected by flow cytometry after naive CD4^+^ T cells were transfected with pCMV-α-SNAP or pCMV (*n* = 4). (**J**) Western blot assay indicated the expression of NF-κB p65 in naive CD4^+^ T cells transfected with pCMV-α-SNAP or pCMV. Data are presented as individual values and represent the mean ± SD. *P < 0.05, **P < 0.01, ***P < 0.001 versus nondiabetic controls by 1-way ANOVA in **C**–**E** and **G** and 2-tailed Student’s *t* test in **H** and **I**. Representative of 3 experiments.

To explore the potential mechanism by which macrophage-derived exosomes regulate Treg differentiation, a proteomic analysis that assessed the protein composition of the exosomes isolated from BMDMs was performed. We conducted volcano plot and heatmap analysis to visualize the patterns of differential protein expression, which clearly delineated distinct expression profiles between the β-catenin^fl/fl^ exosomes and β-catenin^M-KO^ exosomes (Figure 5, A and B). Among these differentially expressed proteins, α-SNAP was reported to be essential for Treg differentiation (31). Next, we validated the expression of *Napa* in the livers, which codes for α-SNAP. qRT-PCR analysis revealed that the expression of *Napa* in the livers was greatly increased after APAP or D-GalN/LPS injection (Figure 5C). Importantly, myeloid β-catenin deficiency decreased *Napa* expression in APAP- and D-GalN/LPS–injured livers (Figure 5D). Consistently, the expression of *Napa* was markedly reduced in β-catenin^M-KO^ BMDMs compared with β-catenin^fl/fl^ BMDMs (Figure 5E). Moreover, we evaluated the α-SNAP protein levels in BMDM exosomes by Western blot. Consistent with these changes, the α-SNAP expression was decreased in β-catenin^M-KO^ BMDM–derived exosomes compared with β-catenin^fl/fl^ BMDM–derived exosomes (Figure 5F).

To further verify macrophage-derived exosomal α-SNAP regulates Treg differentiation, we overexpressed α-SNAP in RAW264.7 macrophages through transfection with α-SNAP plasmid (Supplemental Figure 3B). We found that the Foxp3^+^ Treg population was markedly increased following α-SNAP overexpression (Figure 5G). To further indicate that macrophage β-catenin modulates Treg differentiation via exosomal α-SNAP, we overexpressed α-SNAP in β-catenin^M-KO^ BMDMs (Supplemental Figure 3C). Then, the exosomes from β-catenin^M-KO^ BMDMs+pCMV and β-catenin^M-KO^ BMDMs+pCMV-α-SNAP were collected to coculture with naive CD4^+^ T cells. We found that α-SNAP overexpression in BMDMs reversed the reduction in Treg differentiation caused by β-catenin deficiency (Figure 5H). Similarly, the overexpression of α-SNAP in naive CD4^+^ T cells also markedly increased the proportion of Foxp3^+^ Tregs (Figure 5I).

Recent reports have shown that NF-κB was inhibited by α-SNAP restriction, which can cause a severe block in Foxp3^+^ T cell development in mice (31). In addition, NF-κB has been shown to be necessary for Treg development and function (32–34). To determine whether the expression of NF-κB p65 was affected by α-SNAP overexpression in naive CD4^+^ T cells, we extracted the protein from T cells. As expected, α-SNAP overexpression in naive CD4^+^ T cells markedly enhanced NF-κB p65 expression (Figure 5J). Collectively, these results indicated that macrophage β-catenin modulates Treg differentiation via exosomal α-SNAP.

## Discussion

Macrophages and Tregs are key players in initiating and shaping liver immune responses, which play central roles in injury recognition and homeostasis maintenance in acute liver injury. However, the mechanisms by which hepatic macrophages regulate Tregs in acute liver injury have not been fully elucidated. In this study, we documented that macrophage β-catenin was markedly upregulated and hepatic Treg proportion was increased in acute liver injury. Importantly, β-catenin was upregulated only in infiltrating macrophages but not in resident Kupffer cells. Furthermore, we showed that disruption of myeloid β-catenin promoted inflammatory cell infiltration and hepatocyte apoptosis and decreased the Treg proportion during acute liver injury. Mechanistically, macrophage β-catenin regulated Treg differentiation by modulating the expression of exosomal α-SNAP. Collectively, these findings highlight the importance of macrophage β-catenin in hepatic Treg proportion and liver inflammation and provide evidence and rationale for therapies targeting β-catenin in macrophages.

Accumulating evidence demonstrates that hepatic macrophages and Tregs play an important role in modulating inflammatory liver diseases (3, 35). As the first line of defense against pathogens such as bacteria and viruses, macrophages can be roughly divided into 2 populations because of the heterogeneity in the liver, comprising both liver-resident Kupffer cells and infiltrating macrophages with considerable functional plasticity (3, 4). Moreover, macrophages are highly plastic and adapt their phenotype according to the signals derived from the hepatic microenvironment, which explains their manifold and even opposing functions during progression and regression of injury (3). Previous studies showed that depletion of either population or both populations of liver macrophages could delay liver repair and histological recovery in acute liver injury (36). However, it has also been reported that infiltrating macrophages and resident Kupffer cells display different ontogeny and functions in acute liver injury (37). Indeed, Kupffer cells are reduced in APAP-induced liver injury; in contrast, infiltrating macrophages become the predominant macrophage subset at the necroinflammatory phase (24 hours postchallenge) (37, 38). In keeping with these studies, our results showed that upregulation of β-catenin expression was observed only in infiltrating macrophages but not in resident Kupffer cells. These findings further support the notion that liver macrophages display a remarkable heterogeneity in response to microenvironmental signals during acute liver injury. Previous studies have demonstrated that the active macrophage is tightly connected to Treg homeostasis. Moreover, the proinflammatory macrophages have been identified to cause Th17/Treg imbalance (39). Meanwhile, the higher proportion of Tregs was found in patients with acute hepatitis A (40). Similarly, the present study indicated that hepatic Treg proportion was also increased in APAP- and D-GalN/LPS–induced acute liver injury. The increased proportion of hepatic Tregs may be required for compensation of the liver to maintain essential physiological functions. Intriguingly, in this study, we demonstrated that the increase of Treg proportion in the liver was regulated by macrophage β-catenin during acute liver injury. These findings emphasize the relevance of macrophages or Tregs as an effective immunotherapeutic option for liver diseases.

Various signaling pathways have been implicated in the maintenance of liver immune homeostasis, including β-catenin signaling. The β-catenin pathway is mostly inactive in the healthy liver. However, it can be reactivated during cell renewal and regenerative processes, as well as in certain pathological conditions (41). Indeed, the expression of β-catenin in the liver is increased in acute liver injury, which is essential for liver injury repair (26, 28, 42, 43). In keeping with these results, our data revealed that the β-catenin expression in hepatic infiltrating macrophages was markedly upregulated. Additionally, the recently described role of the β-catenin pathway in regulating immune cell infiltration renewed the interest, given its potential impact on responses to immunotherapy strategies (24). Pro-inflammatory macrophages could hinder hepatic Treg differentiation in liver injury, but activation of β-catenin signals can facilitate M2 macrophage polarization (44, 45). Besides, previous studies showed that macrophage Wnt/β-catenin signaling contributed to liver functional compensation during acute liver injury (46). Consistently, the present study demonstrated that the blockade of macrophage β-catenin decreased the hepatic Treg proportion and exacerbated APAP- and D-GalN/LPS–induced liver damage, which highlighted the importance of macrophage β-catenin during acute liver injury. However, it remains unclear how APAP leads to activation of β-catenin in macrophages. It has been reported that in response to inflammatory injury, lung endothelial cells release the Wnt signaling modulator Rspondin3, which activates β-catenin signaling in lung interstitial macrophages during acute lung injury (47). Moreover, the Wnt signaling in liver endothelial cells was activated during acute liver injury, which could affect liver cell proliferation (46). These studies raise the possibility that macrophage β-catenin may be activated by the secretion of Wnt signal from liver endothelial cells during APAP-induced acute liver injury. Further work is needed to elucidate the precise mechanism of macrophage β-catenin activation during acute liver injury.

Although β-catenin was reported to play a role in macrophage migration (48), our results did show that increased CD11b^+^ macrophages and MPO^+^ neutrophils were detected in livers of β-catenin^M-KO^ mice in 2 independent acute liver injury models. A possible explanation is that signals derived from the hepatic microenvironment during liver injury are complex and these signals integrally regulate the recruitment of macrophages. Moreover, a previous study (49) and our current data demonstrated that myeloid β-catenin knockout aggravated liver inflammation and damage. In this case, the increased inflammatory cytokines and chemokines contributed to the infiltration of monocytes into the liver, which gave rise to large numbers of inflammatory monocyte-derived macrophages. Additionally, macrophages, acting as sentinel cells, reside in tissues and initiate neutrophil recruitment by controlling and inducing various processes, such as increasing permeability of local blood vessels and the release of chemokines (50, 51), during tissue inflammation. Moreover, our present data showed that the β-catenin^M-KO^ mouse liver was accompanied by the augmented infiltration of CD11b^+^ macrophages after liver injury. Therefore, the increased MPO^+^ neutrophils in livers of β-catenin^M-KO^ mice might be mainly caused indirectly by the β-catenin disruption in macrophages.

Macrophage-derived exosomes are shown to play an important role in acute liver injury (52, 53). Our study also indicated that LPS-stimulated macrophage-derived exosomes could be taken up by naive CD4^+^ T cells and regulate Treg differentiation. Meanwhile, GW4869 treatment decreased the proportion of Foxp3^+^ Tregs, further indicating that exosomes are the vital medium of communication between macrophages and T cells. Next, we further explored the potential mechanism of the macrophage-derived exosomes regulating the Treg differentiation by exosomal mass spectrometry–based proteomics. Our results showed that the α-SNAP expression was markedly downregulated after β-catenin was blocked. This finding is in agreement with a recent report that demonstrated a crucial role of α-SNAP in Foxp3^+^ Treg differentiation in vivo as well as in vitro (31). Moreover, we validated the expression of α-SNAP in vivo and in vitro and found that macrophage β-catenin–specific knockout downregulated the α-SNAP level. The NF-κB pathway was identified as an important signaling for Foxp3^+^ T cell development regulated by α-SNAP (31). In agreement with previous findings, our results indicated that α-SNAP overexpression markedly enhanced the expression of NF-κB p65. However, further mechanistic work is needed to fully understand whether the reduction of Tregs is the main reason for the exacerbation of acute liver injury in β-catenin^M-KO^ mice and how macrophage β-catenin regulates the expression of exosomal α-SNAP. Previous studies from other groups and our own have demonstrated that β-catenin signaling facilitates M2 polarization both in vitro and in various in vivo models and thus regulates the progression of various diseases, including tissue inflammation, fibrosis, and tumor (8, 45, 54). Meanwhile, macrophage β-catenin was shown to play an important role in regulating the NLRP3-driven inflammatory response through crosstalk with other signaling pathways (6, 49). In the current study, our findings reveal that macrophage β-catenin modulates its exosomal α-SNAP and influences Treg differentiation during acute liver injury.

Previous studies reported that monocyte-sourced exosomes from blood samples of patients activated macrophages and increased pro-inflammatory cytokines in acute lung injury (55). Similarly, Pei et al. observed a significant increase in the levels of exosomal SLP adaptor and CSK interacting membrane protein (SCIMP) in bronchoalveolar lavage fluid and serum of patients with pneumonia. They demonstrated that exosomal SCIMP regulated communication between macrophages and neutrophils in pneumonia (56). In addition, macrophage-derived exosomal aminopeptidase N from the plasma of patients aggravated sepsis-induced acute lung injury by regulating necroptosis of lung epithelial cells (57). Although we did not directly validate the signaling pathway of macrophage-sourced exosomal α-SNAP in patients with acute liver injury, our data in vivo and in vitro demonstrate the communication between hepatic macrophages and Tregs via the exosome pathway. The aforementioned findings suggest that circulating exosomes might exert a key role in regulating cell-to-cell communication by releasing proteins, mRNAs, miRNAs, and lncRNAs.

In summary, we investigated the association between macrophage β-catenin and hepatic Treg proportion in APAP- and D-GalN/LPS–induced acute liver injury and identified a regulatory mechanism of cell-cell communication between hepatic macrophages and Tregs during acute liver injury. We demonstrated that macrophage β-catenin influenced Treg proportion by regulating the exosomal α-SNAP level. Although we cannot rule out that macrophage β-catenin activation may regulate Treg production through other pathways, our findings add a non-negligible mechanism for the crosstalk between macrophages and CD4^+^ T cells, providing insights into the pathophysiology of acute liver injury.

## Methods

### Sex as a biological variable.

Sex was not considered as a biological variable.

### Animals.

The floxed β-catenin (β-catenin^fl/fl^) mice (Jackson Laboratory) and the mice expressing Cre recombinase under the control of the lysozyme 2 (Lyz2) promoter (LysM-Cre; Jackson Laboratory) were used to generate myeloid-specific β-catenin knockout (β-catenin^M-KO^) mice. In brief, 2 steps were used to generate β-catenin^M-KO^ mice. First, a homozygous *loxP*-flanked β-catenin mouse was mated with a homozygous Lyz2-Cre mouse to generate the F_1_ mice that were heterozygous for a *loxP*-flanked β-catenin allele and heterozygous for the Lyz2-Cre. Next, these F_1_ mice were backcrossed to the homozygous *loxP*-flanked β-catenin mice, resulting in the generation of β-catenin^M-KO^ (25% of the offspring), which were homozygous for the *loxP*-flanked β-catenin allele and heterozygous for the Lyz2-Cre allele (Supplemental Figure 2). Mouse genotyping was performed by using a standard protocol with primers described in the JAX genotyping protocols database (https://www.jax.org/protocol/search).

Animals at 6–8 weeks of age were used in all experiments. The mice were bred in a standard environment with a 12-hour light/12-hour dark cycle.

### Mouse acute liver injury model.

For the APAP-induced acute liver injury, APAP solution was freshly prepared for each experiment by dissolving APAP in PBS at a concentration of 10 mg/mL and warmed to 40°C. Mice were fasted for 14 hours and then given PBS (i.p.) or APAP (300 mg/kg, i.p.). Mice were sacrificed for collecting serum and liver tissues at 24 hours after APAP injection.

For the D-GalN/LPS–induced acute liver injury, the mice were fasted for 14 hours and then injected i.p. with a dose of 30 μg/kg LPS plus 600 mg/kg D-GalN. Mice were sacrificed for collecting serum and liver tissues at 5 hours after D-GalN/LPS injection.

### Histology, immunohistochemistry, and immunofluorescence staining.

Fresh liver tissues were fixed in 4% paraformaldehyde, embedded in paraffin, and sectioned into 5 μm sections. Liver sections were stained with H&E. Liver monocyte and neutrophil infiltration was detected by immunohistochemistry staining, using primary rabbit anti-mouse CD11b Ab (ab133357, Abcam) and primary rabbit anti-mouse MPO Ab (14569S, Cell Signaling Technology), respectively. Cleaved caspase-3 was detected using primary rabbit anti-mouse cleaved caspase-3 Ab (9662S, Cell Signaling Technology). The secondary biotinylated goat Anti-Rabbit IgG (5220-0336, SeraCare) was used for immunohistochemistry staining. The average number of positive cells was quantified by analyzing at least 10 random high-power fields per animal, with Image-Pro Plus software.

CD68 and β-catenin double-positive macrophages were identified by primary rabbit anti-mouse CD68 (ab125212, Abcam) and rabbit monoclonal Ab β-catenin (8480, Cell Signaling Technology), respectively. CD11b and active β-catenin double-positive macrophages were identified respectively by primary rabbit anti-mouse CD11b (AF6396, Beyotime) and rabbit monoclonal Ab active β-catenin (non-phospho-Ser33/37/Thr41) (8814, Cell Signaling Technology). Images were captured using an inverted Olympus fluorescence microscope.

### Hepatocellular function assay.

Serum ALT and AST levels, which are an indicator of hepatocellular injury, were measured by an automated chemical analyzer (Olympus Automated Chemistry Analyzer AU5400).

### TUNEL assay.

Apoptotic cells were identified with an apoptosis detection kit (S7110, EMD Millipore). Cells with nuclear positive staining by fluorescent antibodies for DNA fragmentation were visualized directly by fluorescence microscopy and counted.

### Flow cytometry analysis.

We initially anesthetized mice with 0.1 mL 1% pentobarbital to prepare nonparenchymal liver cells. Then 20 mL preinfusion solution (deionization Hanks solution containing 0.5% phosphatase inhibitor) and 20 mL postinfusion solution (Hanks solution containing 0.5% phosphatase inhibitor, 0.002% DNase I, 0.05% collagenase IV, 1% BSA) were perfused from the inferior vena cava to liver, respectively. The flow rate was 5 mL/min. Perfused livers were dissected and teased through a 200-mesh filter screen (LD-3084, LDBIO). The hepatocytes were removed by centrifuging at 50*g* for 5 minutes. The collected supernatant was centrifuged at 400*g* for 5 minutes, and red blood cell lysis buffer was used to lyse red blood cells. Then, nonparenchymal cells were obtained by centrifugation at 400*g* for 5 minutes again.

For analysis of the mouse Treg population in the liver, nonparenchymal cells were incubated with fluorescein isothiocyanate (FITC)/anti-CD3 (11-0032-82, eBioscience), allophycocyanin (APC)/anti-CD4 (17-0041-82, eBioscience), and phycoerythrin-cyanine7 (PE-Cy7)/anti-CD25 (25-0251082, eBioscience) Abs for 30 minutes at 4°C in the dark. After extensive washing, cells were fixed and permeabilized with Foxp3 Fixation/Permeabilization working solution according to the manufacturer’s instruction (00-5523-00, eBioscience) and incubated with PE/anti-Foxp3 Ab (12-4771-82, eBioscience) for 30 minutes at room temperature (RT) in the dark.

To detect the expression of β-catenin/active β-catenin (non-phospho-Ser33/37/Thr41) in hepatic macrophages, nonparenchymal cells were incubated with FITC/anti-CD11b (557396, BD Biosciences) and PE-Cy7/anti-F4/80 (123114, BioLegend) Abs for 30 minutes at 4°C in the dark. After extensive washing, cells were fixed, permeabilized for 30 minutes at RT, and blocked for 30 minutes at RT with PBS containing 5% BSA. Then the cells were incubated with rabbit monoclonal Ab β-catenin (8480, Cell Signaling Technology)/active β-catenin (non-phospho-Ser33/37/Thr41) (8814, Cell Signaling Technology) Ab for 1 hour and Goat Anti-Rabbit IgG/APC Ab (bs-0295G-APC, Bioss) for 30 minutes at RT.

To analyze the Treg (Foxp3^+^) proportion of naive CD4^+^ T cells in the coculture experiments in vitro, cells were stained with APC/anti-CD4 Ab for 30 minutes at 4°C in the dark. After extensive washing, cells were fixed and permeabilized with Foxp3 Fixation/Permeabilization working solution according to the manufacturer’s instruction and incubated with PE/anti-Foxp3 Ab for 30 minutes at RT in the dark. Stained cells were measured with FACSAria III flow cytometer (BD Biosciences).

### Western blot analysis.

Protein was extracted from liver tissue or cells with RIPA lysis buffer containing 1% PMSF and 1% proteinase and phosphatase inhibitor cocktails (MilliporeSigma). Then, protein concentrations were determined by BCA Protein Assay Kit (Thermo Fisher Scientific). Equal amounts of proteins (25–50 μg) were separated by 12% SDS-PAGE and electrotransferred onto a PVDF membrane (Bio-Rad). The membrane was blocked with 5% dry milk and 0.1% Tween 20 TBS buffer (pH 7.4) for 1 hour and incubated overnight at 4°C in primary Abs, followed by the corresponding secondary Abs. The used primary Abs included β-catenin (A19657, A11932, ABclonal), β-tubulin (10068-1-AP, Proteintech), GAPDH (AC001, ABclonal), p–β-catenin (5651, Cell Signaling Technology), β-Actin (AC038, ABclonal), TSG101 (ab125011, Abcam), CD9 (A19027, ABclonal), CD63 (ab217345, Abcam), α-SNAP (MA5-36194, Invitrogen), and NF-κB p65 (8242, Cell Signaling Technology). The used secondary Abs included Goat anti-Rabbit IgG (H+L) (AS014, ABclonal) and Goat anti-Mouse IgG (H+L) (AS003, ABclonal). The membrane was imaged by ECL Senicapture gel image acquisition software (Peiqing).

### qRT-PCR analysis.

Liver tissues or cell cultures were lysed with TRIzol (Invitrogen), and then RNA was purified according to the manufacturer’s instructions. RNA (0.5 μg) was reverse-transcribed to cDNA by the PrimeScript RT reagent kit with gDNA Eraser (Takara Biotechnology Co. Ltd). cDNA was quantified by real-time PCR using the Hieff qPCR SYBR Green Master Mix (YEASEN) and CFX 96 Detection System (Bio-Rad). Primer sequences used for the amplification of *Ctnnb1*, *Napa*, and *Gapdh* are shown in Supplemental Table 1. Target gene expressions were calculated by their ratios to the housekeeping gene *Gapdh*.

### Isolation of BMDMs and naive CD4^+^ T cells.

Murine primary BMDMs were isolated, as described (5). In brief, bone marrow cells were harvested from the femurs and tibias of β-catenin^fl/fl^ and β-catenin^M-KO^ mice and cultured in DMEM supplemented with 10% FBS and 20% L929-conditioned medium.

We used EasySep Mouse naive CD4^+^ T cell Isolation Kits (Stem Cell Technologies) to purify naive CD4^+^ T cells from the mouse spleen (purity > 90%).

### Cell culture and coculture system.

In the Treg differentiation assay, 3 × 10^5^ naive CD4^+^ T cells were cocultured with 3 × 10^5^ BMDMs in RPMI 1640 complete medium (MA0548, Meiluncell) containing soluble anti-CD3 (1 μg/mL) (100340, BioLegend), soluble anti-CD28 (1 μg/mL) (102116, BioLegend), and recombinant murine IL-2 (5 μg/mL) (212-12, PeproTech). Cells were harvested for Foxp3 analysis after 3 days.

To verify the role of exosomes, BMDMs were pretreated with 5 μM GW4869 (D1692, MilliporeSigma) or DMSO for 6 hours. Then the pretreated BMDMs were cocultured with naive CD4^+^ T cells for 3 days, during which GW4869 treatment was continuously performed.

For the coculture of exosomes and naive CD4^+^ T cells, 3 × 10^5^ naive CD4^+^ T cells were prestimulated with soluble anti-CD3 (1 μg/mL), soluble anti-CD28 (1 μg/mL), and recombinant murine IL-2 (5 ng/mL) for 3 days. The BMDM exosomes (30 μg/mL) were added to the system, and coculture was continued for 3 days.

### Plasmid and siRNA.

The pCMV-α-SNAP plasmid and empty vector were purchased from MIAOLING Biology. The oligos were purchased from GenePharma, and the sequences of β-catenin siRNA were as follows: 5′-CCAGGUGGUAGUUAAUAAATT-3′ (sense siβ-catenin), 5′-UUUAUUAACUACCACCUGGTT-3′ (antisense siβ-catenin).

### In vitro transfection.

BMDMs (1 × 10^6^/well) were transfected with the pCMV-α-SNAP plasmid or β-catenin siRNA by Lipo8000 transfection reagent (C0533, Beyotime) according to the manufacturer’s instructions. After 48 hours, cells were supplemented with 500 ng/mL LPS for an additional 6 hours.

### Exosome labeling for cellular uptake.

To examine the uptake of BMDM exosomes by naive CD4^+^ T cells in vitro, BMDM exosomes were labeled using a PKH27 red fluorescence labeling kit (MINI26, MilliporeSigma). The labeled BMDM exosomes were then incubated with the naive CD4^+^ T cells at 37°C for 8 hours. The exosome-treated naive CD4^+^ T cells were then fixed and imaged by confocal microscopy (Leica Microsystems).

### Isolation and characterization of BMDM exosomes.

The concentrated medium was performed by differential centrifugation to isolate the exosomes. All centrifugations and procedures were carried out at 4°C to minimize protein degradation. Cells present in the concentrated medium were removed by low-speed centrifugation at 300*g* for 10 minutes. The cleared supernatant was then sequentially centrifuged at 2,000*g* for 10 minutes and 10,000*g* for 30 minutes to remove any remaining cell debris/microvesicles. The solution was subsequently ultracentrifuged at 100,000*g* for 70 minutes to pellet exosomes. The exosomes were resuspended in PBS and stored at –80°C for further use.

Then approximately 5 μL of the exosome suspension sample was applied to carbon-coated copper grids for 10 minutes at RT (24°C) and negatively stained with 2% phosphotungstic acid solution (pH 6.5). The morphology of exosomes was observed by HT7700 transmission electron microscope (Hitachi). Particle size distribution and concentrations were measured by NTA (Particle Metrix). Positive biomarkers (TSG101, CD9, and CD63) were detected by Western blot.

### LC/MS analysis.

The protein extraction and LC/MS analysis were done on protein from BMDM exosomes by DLMBiotech. In brief, the extracted peptides in each sample were analyzed using label-free quantification on an LC/MS online with a Q Exactive Plus mass spectrometer (Thermo Fisher Scientific) coupled to an EASY-nLC 1200 system (Thermo Fisher Scientific). The analysis was operated in a data-dependent acquisition mode. The full mass spectrometry survey scan resolution was set to 70,000 with an automated gain control (AGC) target of 3 × 10^6^ for a scan range of 340–1,800 *m/z* and a maximum injection time of 20 ms. The higher energy collision dissociation fragmentation was performed at a normalized collision energy of 28%. The MS2 AGC target was set to 2 × 10^5^ with a maximum injection time of 50 ms, and the dynamic exclusion was set to 35 seconds.

The MS/MS data were searched against the concatenated target/decoy Mouse UniProt (58) database as of January 1, 2023, with only reviewed and canonical sequences used. MaxQuant (V1.6.6) was then used to infer protein identifications. Label-free quantification and subsequent median normalization of each protein were performed using weighted spectral counting (59).

The mass spectrometry proteomics data have been deposited to the ProteomeXchange Consortium via the PRIDE (60) partner repository with the dataset identifier PXD051425.

### ELISA.

The concentrations of IL-10 were measured with ELISA kits (RK00016, ABclonal) according to the manufacturer’s instructions.

### Statistics.

Statistical analysis was done using 2-tailed Student’s *t* test or 1-way ANOVA. Results were shown as mean ± SD; *P* values < 0.05 were considered statistically significant. All analyses were performed by Prism 7.0 (GraphPad Software).

### Study approval.

All animal protocols were approved by the Animal Care and Use Committee of Wuhan University in China (Permit No: WP20220117). The animals received humane care according to the criteria outlined in the *Guide for the Care and Use of Laboratory Animals* published by the National Institutes of Health (National Academies Press, 2011).

### Data availability.

All nonsequencing data are contained within the Supporting Data Values XLS file. The mass spectrometry proteomics data have been deposited to the ProteomeXchange Consortium via the PRIDE (60) partner repository with the dataset identifier PXD051425.

## Author contributions

RZ and YL performed in vitro and in vivo experiments and data analysis; MZ, BL, and WZ performed in vitro experiments; CL and HH contributed to the study concept and research design; and RZ and CL wrote the manuscript. All authors contributed to the article and approved the submitted version.

## Supplementary Material

Supplemental data

Unedited blot and gel images

Supporting data values

## Version 1 11/19/2024

In-Press Preview

## Version 2 01/09/2025

Electronic publication
