# Supplementary material for: β-Catenin disruption decreases macrophage exosomal α-SNAP and impedes Treg differentiation in acute liver injury
Source: JCI Insight. 2024 Nov 19;10(1):e182515. doi: 10.1172/jci.insight.182515 (PMC11721303; doi:10.1172/jci.insight.182515)
Supplement: Supplemental data [file jciinsight-10-182515-s033.pdf]

Supplementary Materials

β-catenin disruption decreases macrophage exosomal α-SNAP and impedes Treg differentiation in acute liver injury

Ruobin Zong[1, #], Yujie Liu[1, #], Mengya Zhang[1], Buwei Liu[1], Wei Zhang[1], Hankun Hu[2, 3, 4,*], Changyong Li[1, 5, *]

[1] Department of Physiology, Wuhan University TaiKang Medical School (School of Basic Medical Sciences), Wuhan, China.

[2] Department of Pharmacy, Zhongnan Hospital of Wuhan University, Wuhan, Hubei, China.

[3] Hubei Micro-explore Innovative Pharmaceutical Research Co., Ltd, Wuhan, Hubei, China.

[4] Suzhou Organ-on-a-Chip System Science and Technology Co., Ltd, Suzhou, Jiangsu, China.

[5] Xianning Medical College, Hubei University of Science & Technology, Xianning, China.

[#] Ruobin Zong and Yujie Liu have contributed equally to this work.

***Corresponding authors:**

Changyong Li, MD, PhD.

Department of Physiology, School of Basic Medical Sciences, Wuhan University, 115 Donghu Road, Wuhan 430071, China.

Email: lichangyong@whu.edu.cn

Phone number: +86 13886054335

Hankun Hu, MD, PhD.

Department of Pharmacy, Zhongnan Hospital of Wuhan University, School of Pharmaceutical Sciences, Wuhan University, 169 Donghu Road, Wuhan 430071, China.

Email: huhankun@whu.edu.cn

Phone number: +86 13971431601

Supplementary Figure 1:

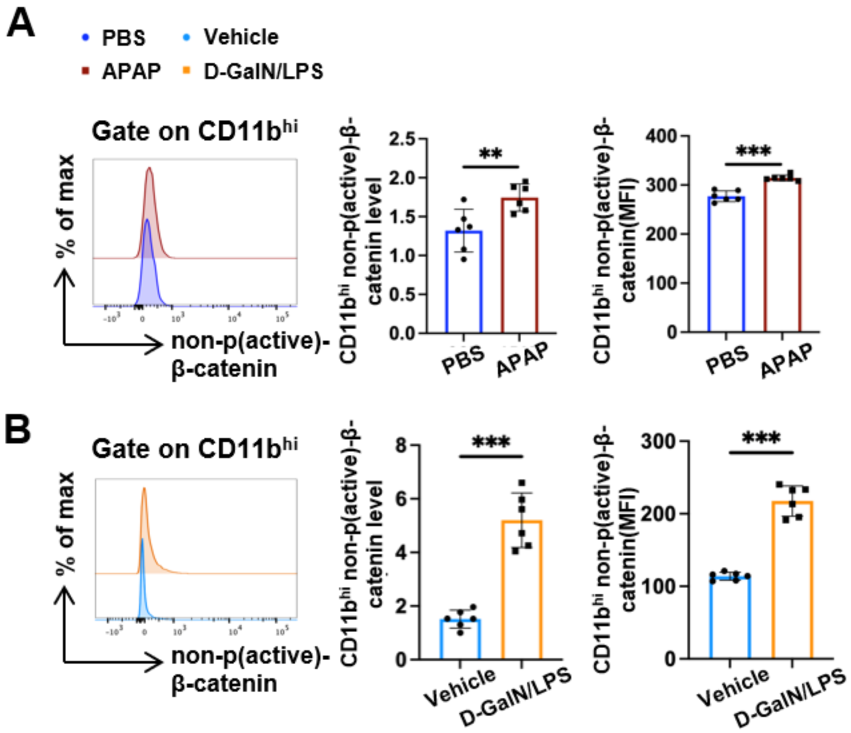

Supplementary Figure 1. Active β-catenin (non-phospho Ser33/37/Thr41) is upregulated in infiltrating macrophages during acute liver injury. For APAP overdose-induced acute liver injury, male mice were i.p. injected with APAP (300 mg/kg BW) or same volume of PBS after overnight fasting and the mice were sacrificed 24 h after injection. For D-GalN/LPS-induced acute liver injury, male mice were intraperitoneally (i.p.) injected with 600 mg/kg BW (body weight) of D-GalN and 30 μg/kg BW of LPS, or same volume of vehicle, and the mice were sacrificed 5 h after injection. (A) The expression of non-p(active)-β-catenin in APAP-induced hepatic macrophages was examined by flow cytometry (n = 6). (B) The expression of non-p(active)-β-catenin in D-GalN/LPS induced hepatic macrophages was examined by flow cytometry (n = 6).

Supplementary Figure 2:

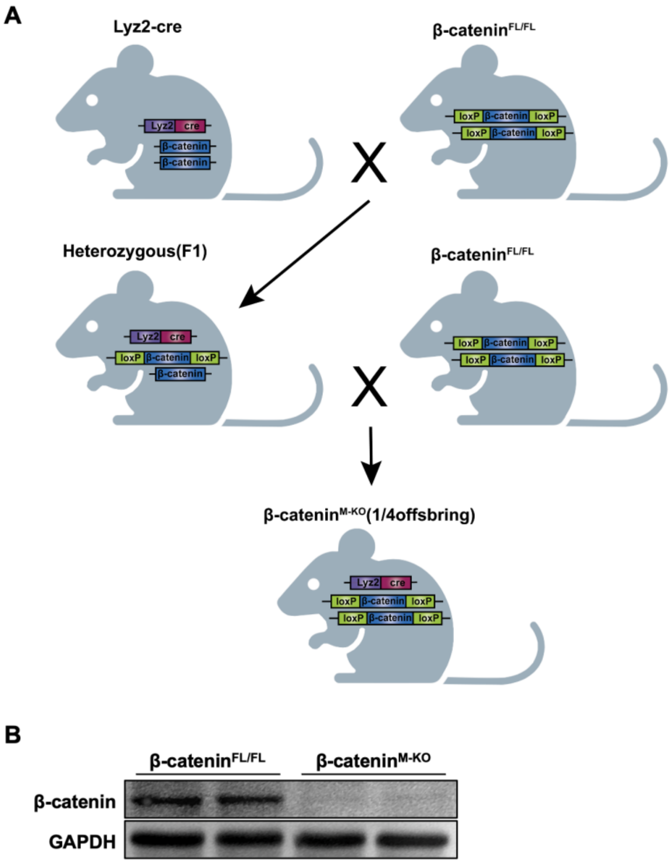

Supplementary Figure 2. Schematic illustration of generation of myeloid-specific β-catenin knockout mice. (A) Schematic diagram of β-catenin$^{M\text{-}KO}$ mouse construction. (B) Western blot analysis of the β-catenin expression in BMDMs.

Supplementary Figure 3:

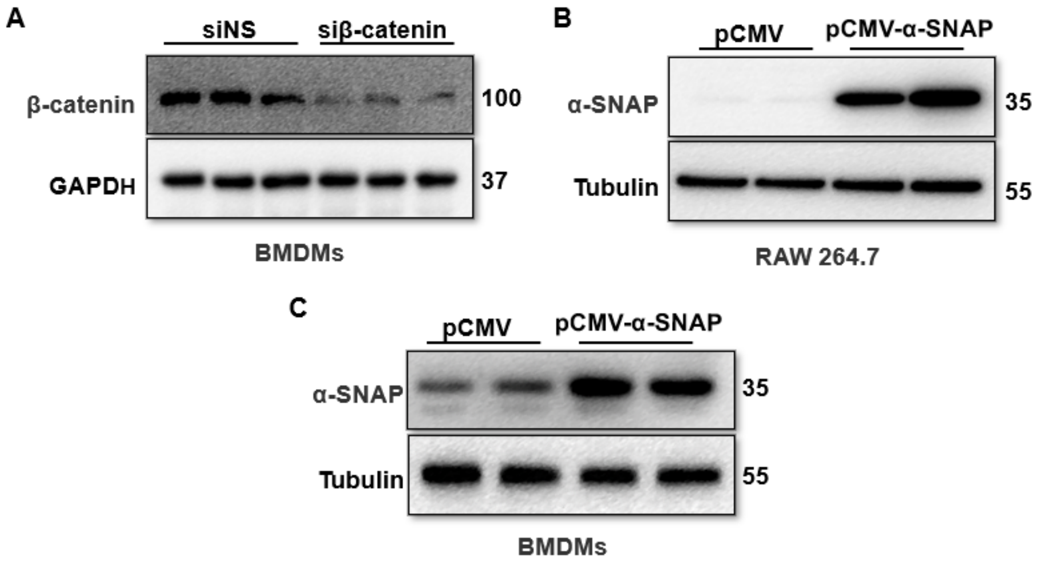

Supplementary Figure 3. Validation of cell transfection. BMDMs were transfected with the β-catenin siRNA or pCMV-α-SNAP plasmid. RAW264.7 were transfected with pCMV-α-SNAP plasmid. (A) Western blot analysis of the β-catenin expression in BMDMs. (B) Western blot analysis of the α-SNAP expression in Raw264.7 cells. (C) Western blot analysis of the α-SNAP expression in BMDMs.

Supplementary Figure 4:

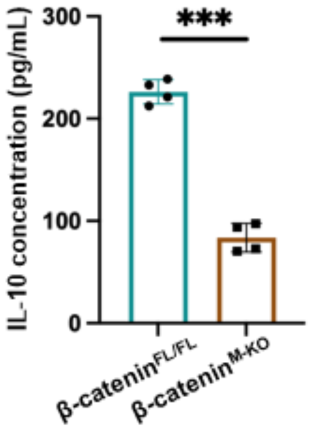

Supplementary Figure 4. The effect of macrophage β-catenin on the immunosuppressive function of Tregs. The naïve $CD4^+$ T cells were co-cultured with BMDMs isolated from β-catenin$^{FL/FL}$ and β-catenin$^{M\text{-}KO}$ mice. The level of IL-10 in Tregs was measured by ELISA (n=4). Values represent means ± SD. ***$p < 0.001$

Supplementary Figure 5:

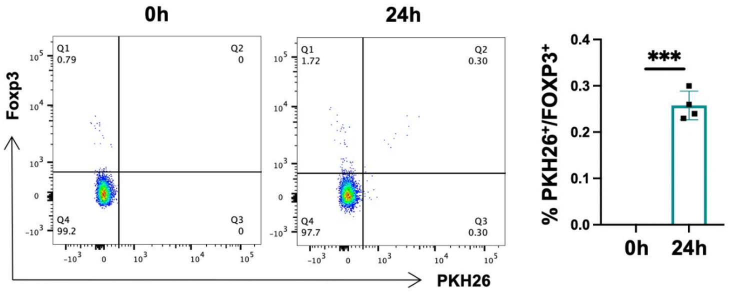

Supplementary Figure 5. Treg cells take up macrophage exosomes. BMDM-exosomes were labeled with PKH26 (red) and then co-cultured with naïve $CD4^+$ T cells for 24h. Then the induction of $Foxp3^+$ Tregs was detected by flow cytometry (n = 4). Values represent means ± SD. ***$p < 0.001$

Supplementary Table 1. The list of primers used in this study.

Gene	Forward primer (5'-3')	Reverse primer (5'-3')
Gapdh	CATCACTGCCACCCAGAAGACTG	ATGCCAGTGAGCTTCCCGTTCAG
Ctnnb1	GTTCGCCTTCATTATGGACTGCC	ATAGCACCCTGTTCCCGCAAAG
Napa	CTGCTTTGTGGACGCTGGCAAT	AGATGTGGTGCTTGGCTGCGAT
