# Supplementary material for: β-Catenin disruption decreases macrophage exosomal α-SNAP and impedes Treg differentiation in acute liver injury
Source: JCI Insight. 2024 Nov 19;10(1):e182515. doi: 10.1172/jci.insight.182515 (PMC11721303; doi:10.1172/jci.insight.182515)
Supplement: Unedited blot and gel images [file jciinsight-10-182515-s034.pdf]

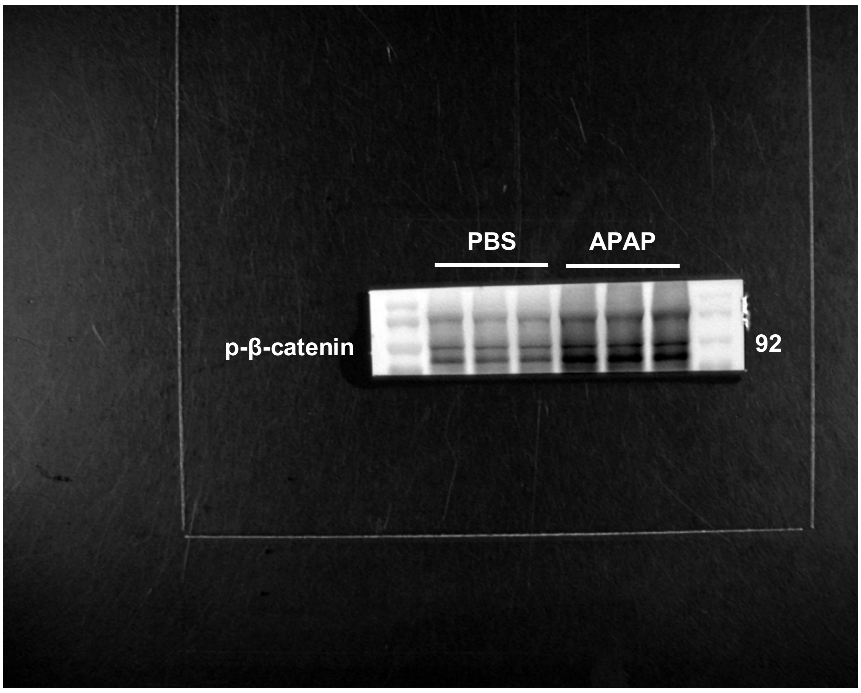

Full unedited gel for Figure 1.A

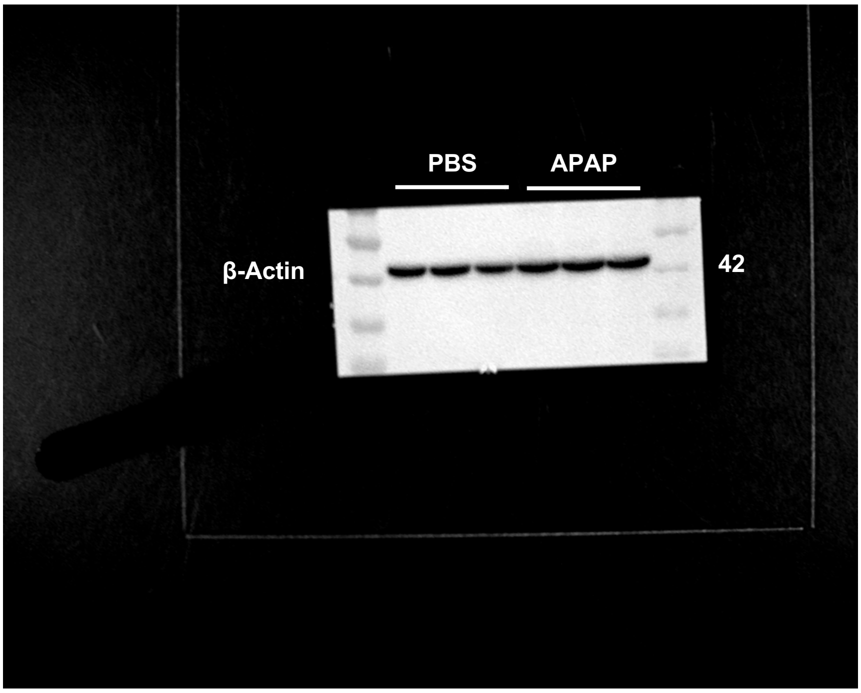

Full unedited gel for Figure 1.A

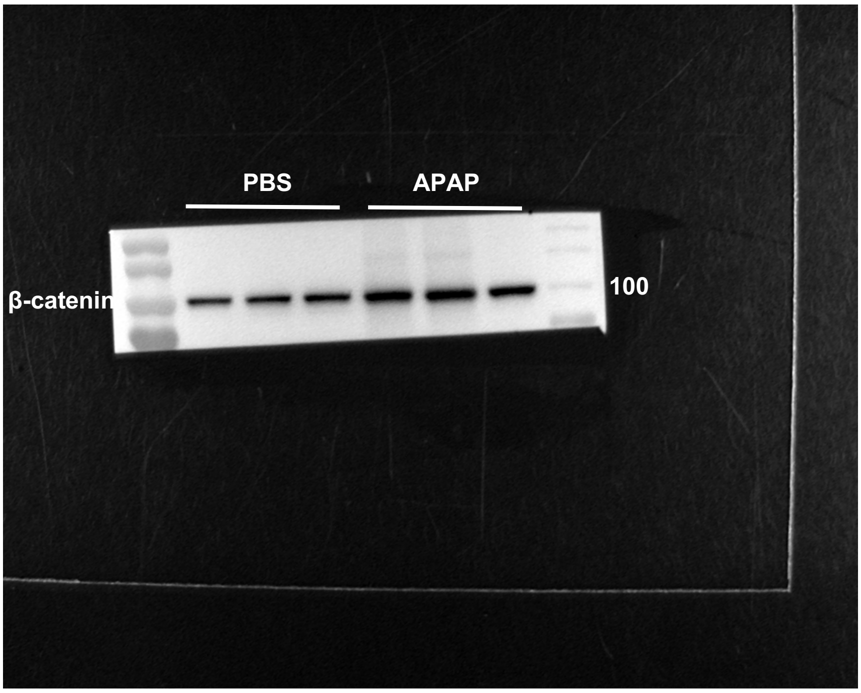

Full unedited gel for Figure 1.A

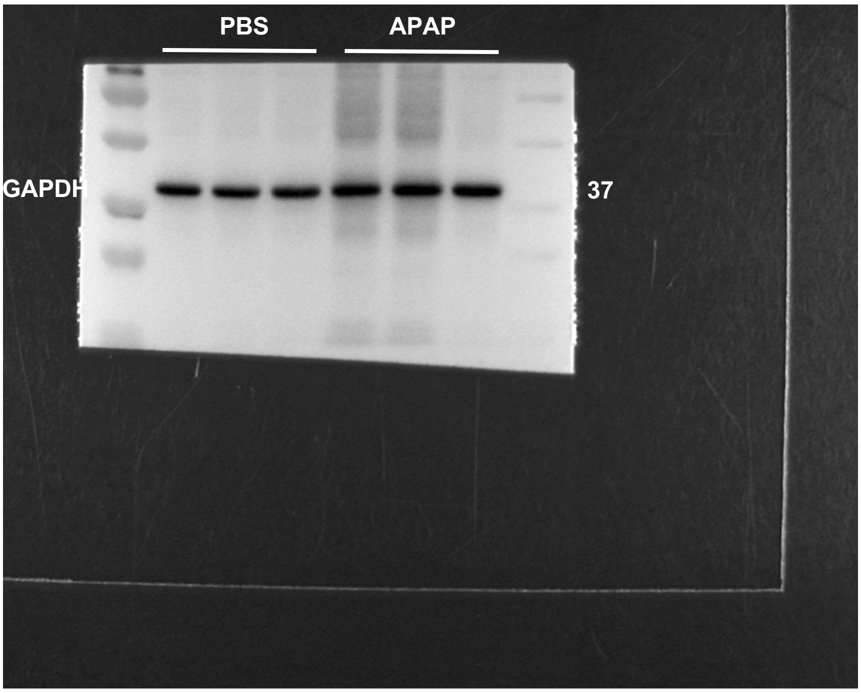

Full unedited gel for Figure 1.A

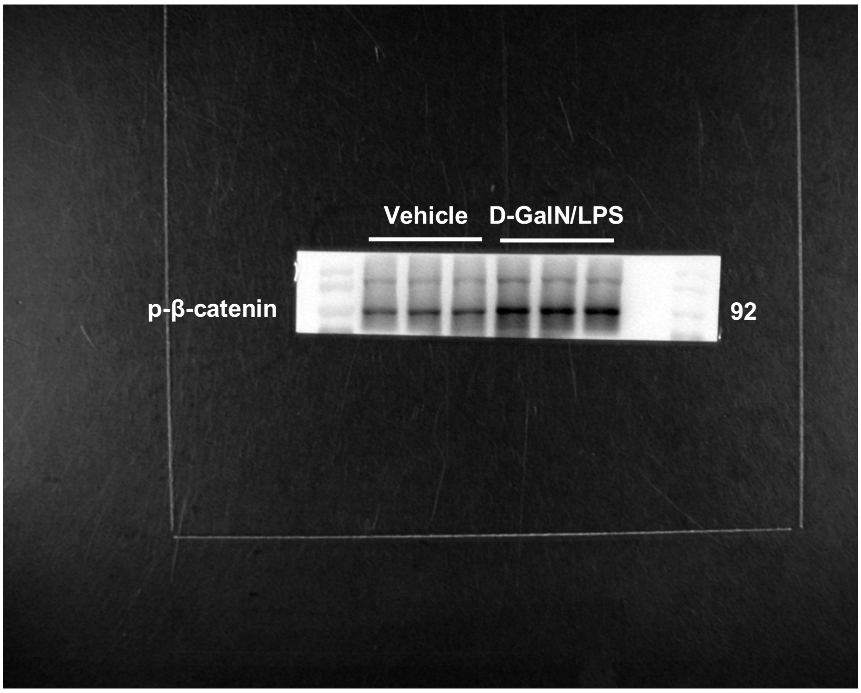

Full unedited gel for Figure 1.A

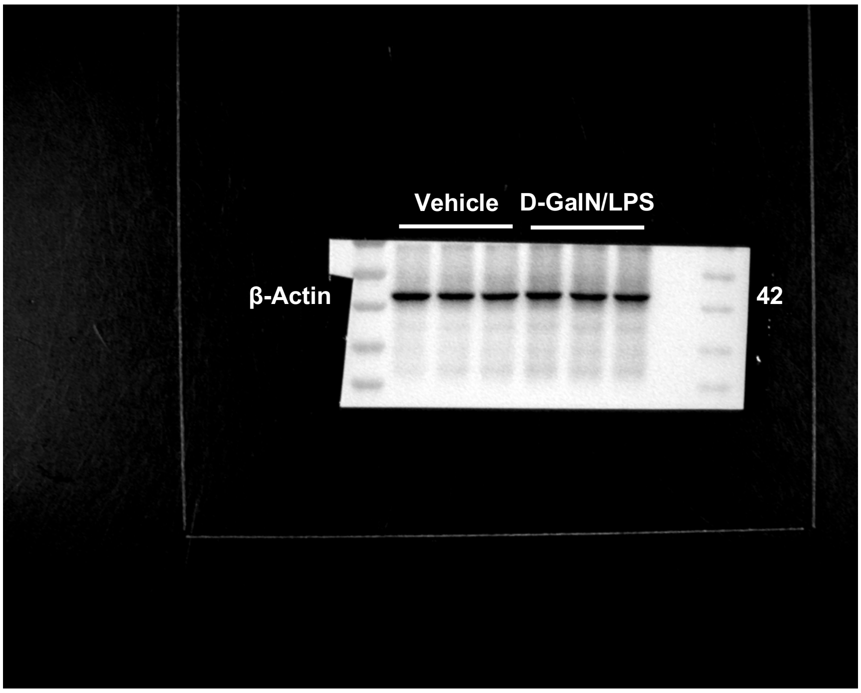

Full unedited gel for Figure 1.A

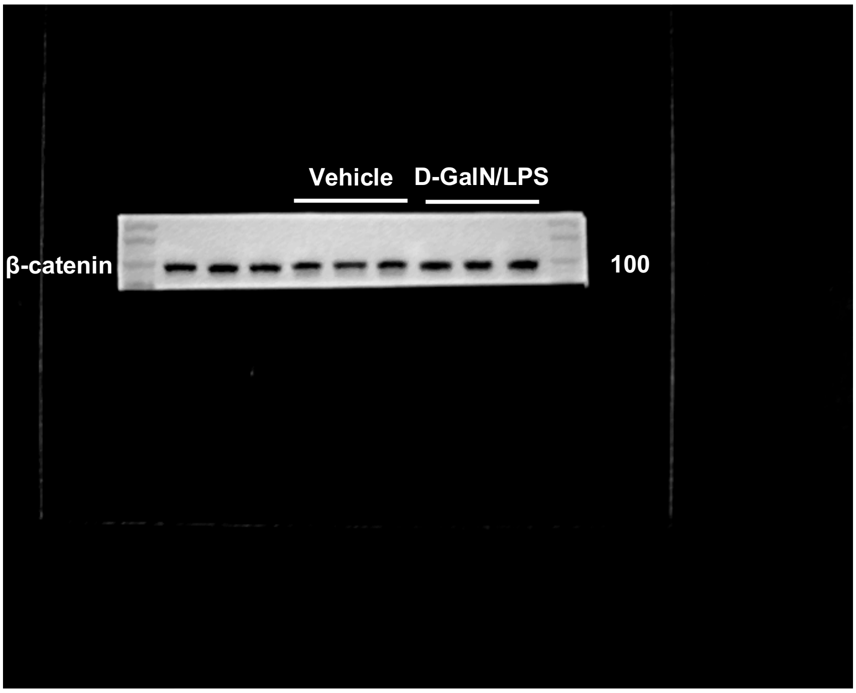

Full unedited gel for Figure 1.A

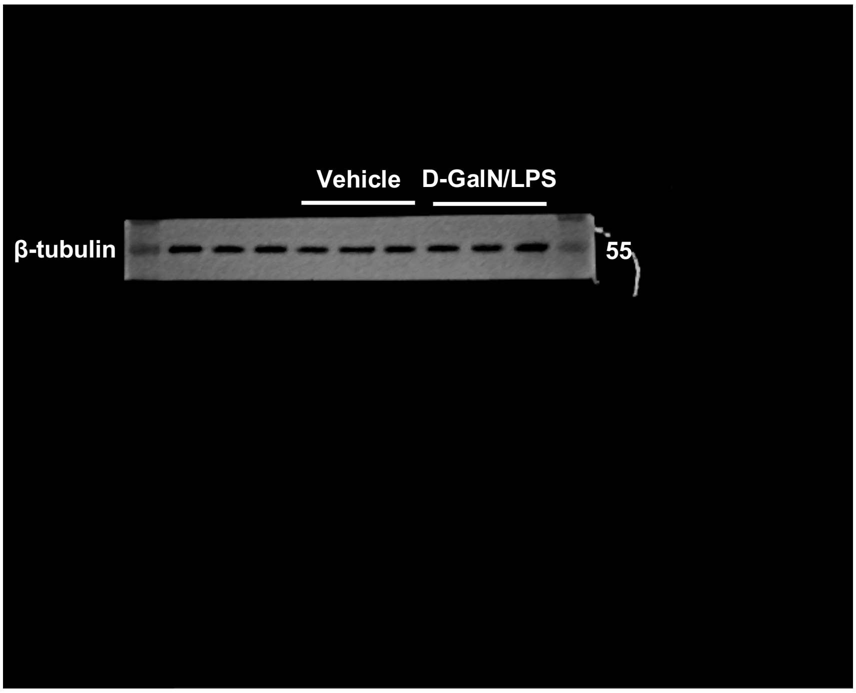

Full unedited gel for Figure 1.A

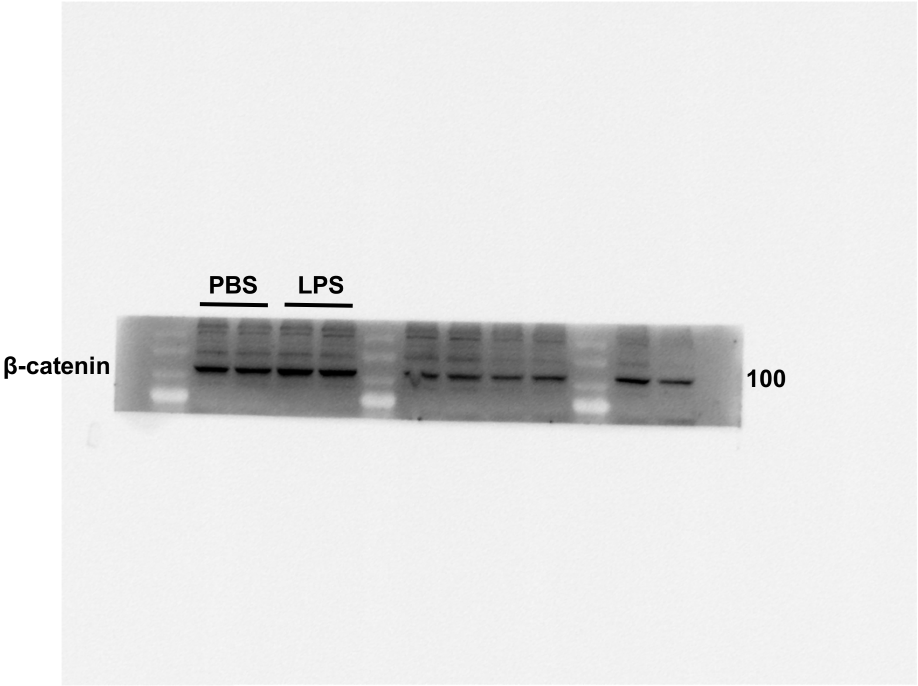

Full unedited gel for Figure 3.A

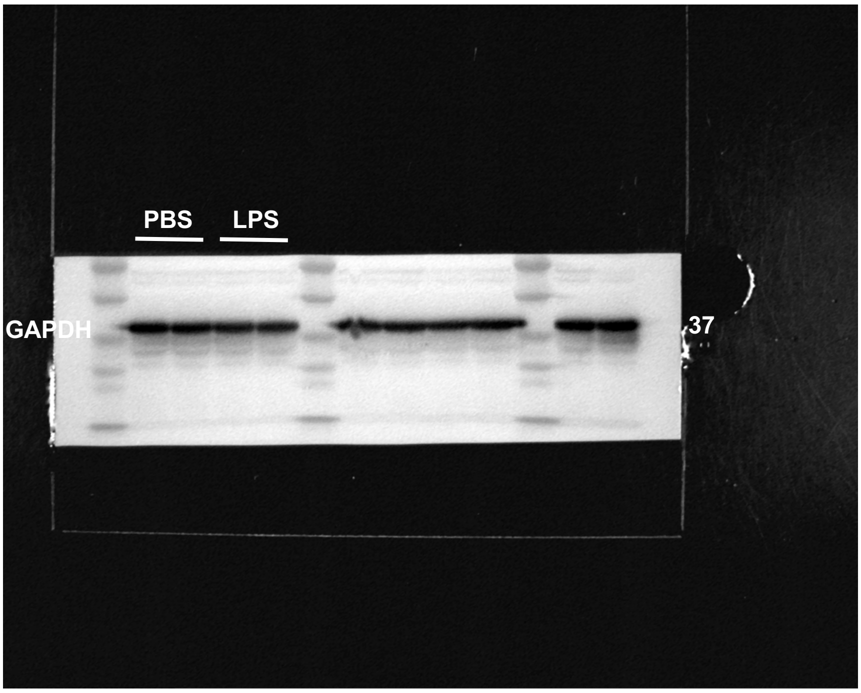

Full unedited gel for Figure 3.A

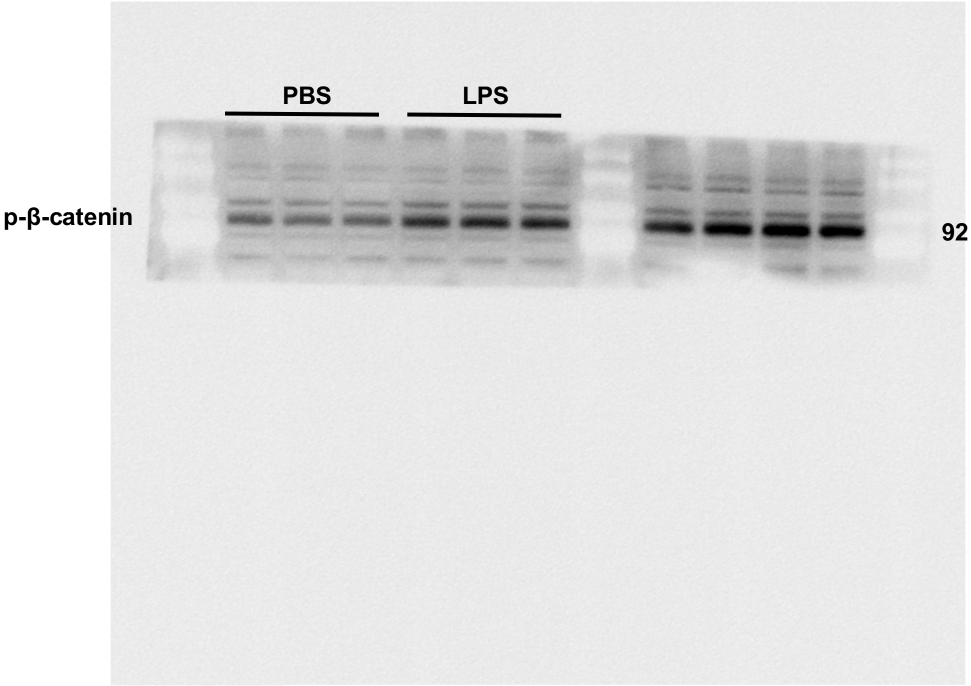

Full unedited gel for Figure 3.A

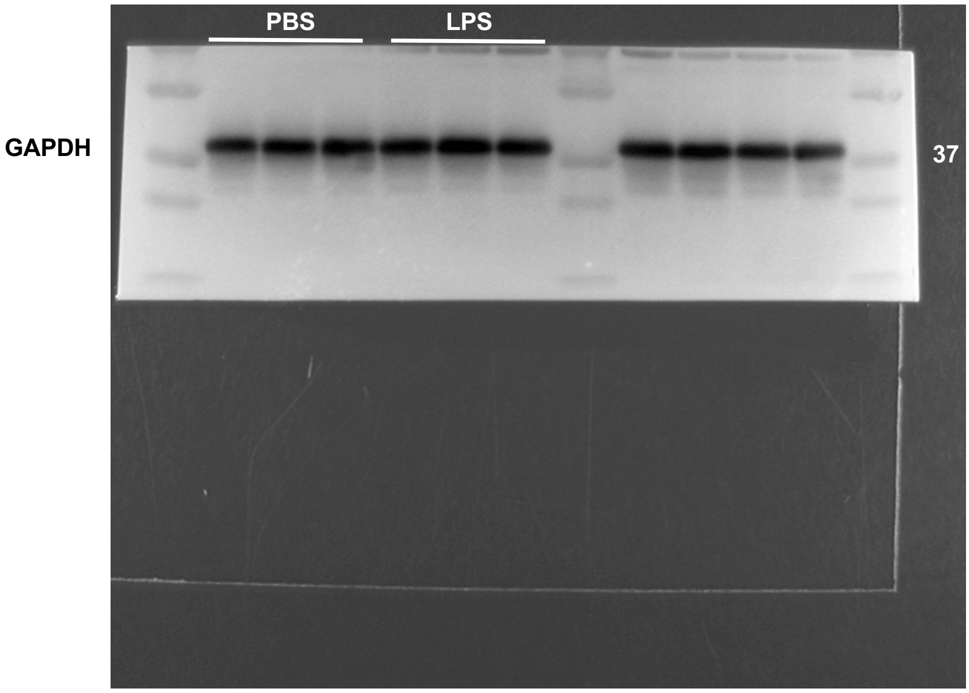

Full unedited gel for Figure 3.A

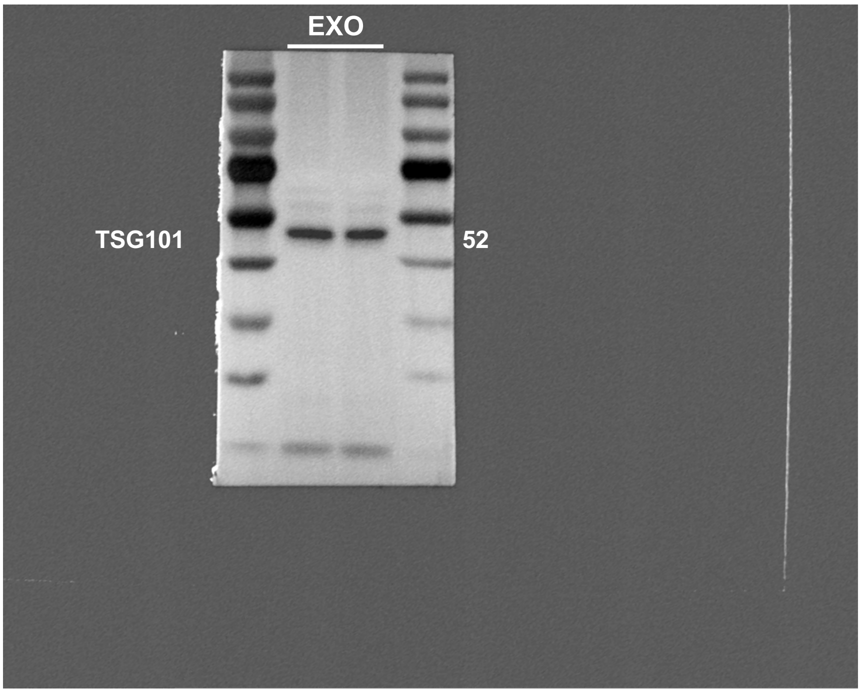

Full unedited gel for Figure 4.D

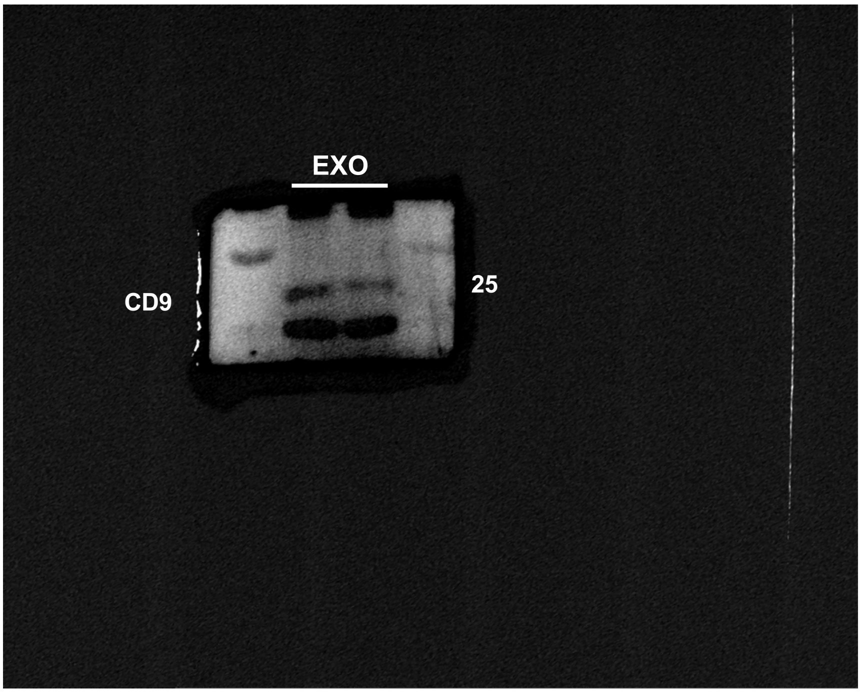

Full unedited gel for Figure 4.D

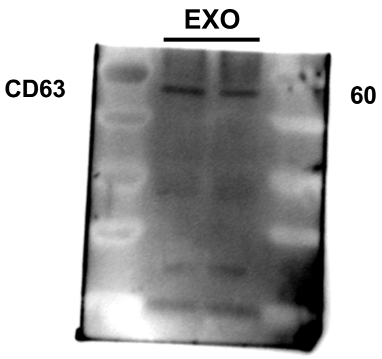

Full unedited gel for Figure 4.D

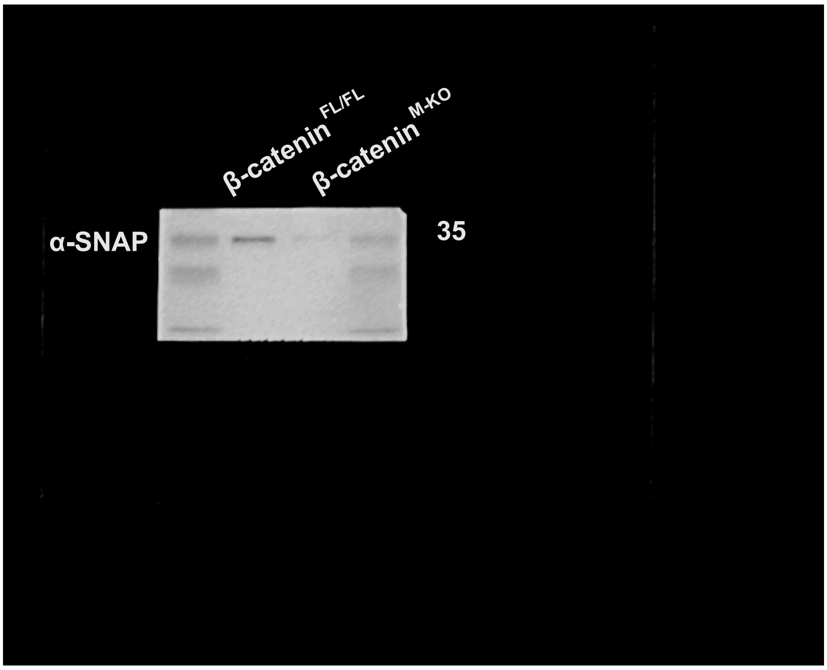

Full unedited gel for Figure 5.F

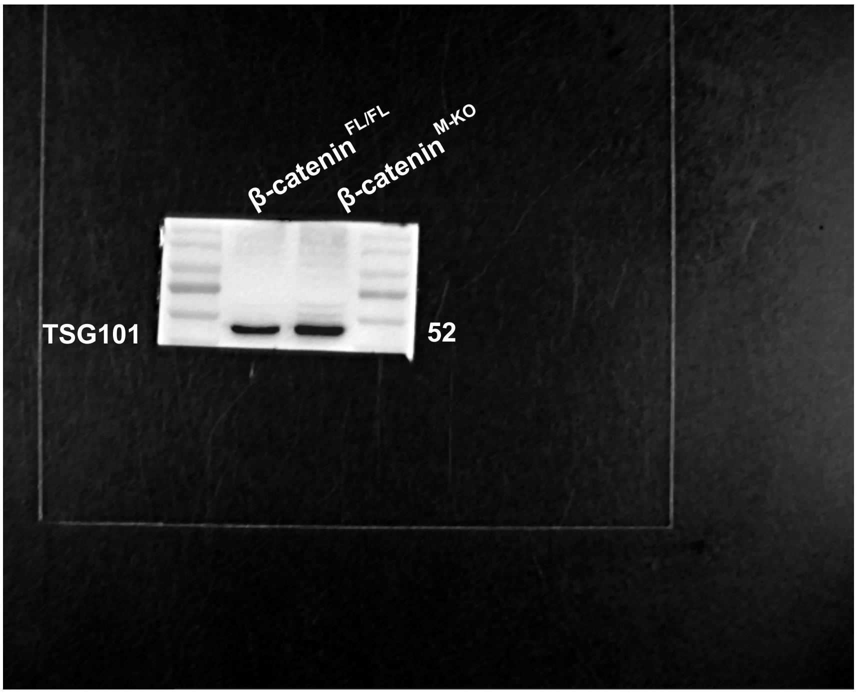

Full unedited gel for Figure 5.F

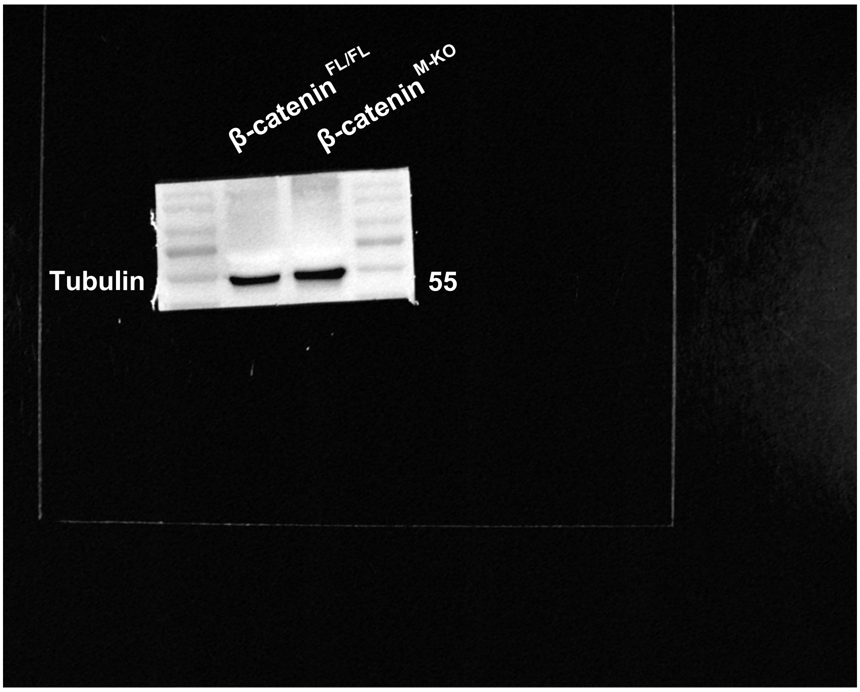

Full unedited gel for Figure 5.F

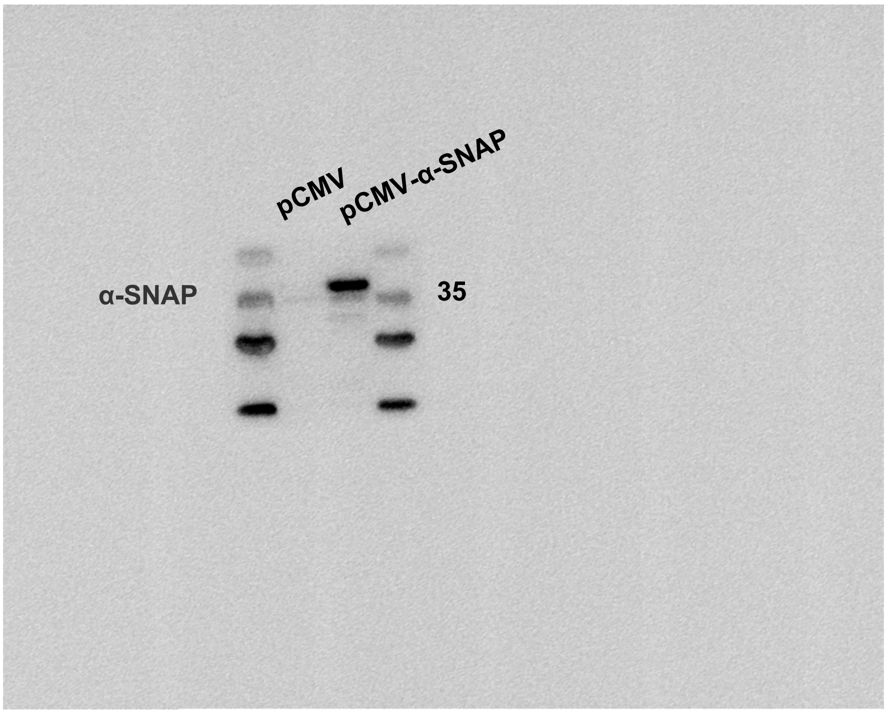

Full unedited gel for Figure 5.I

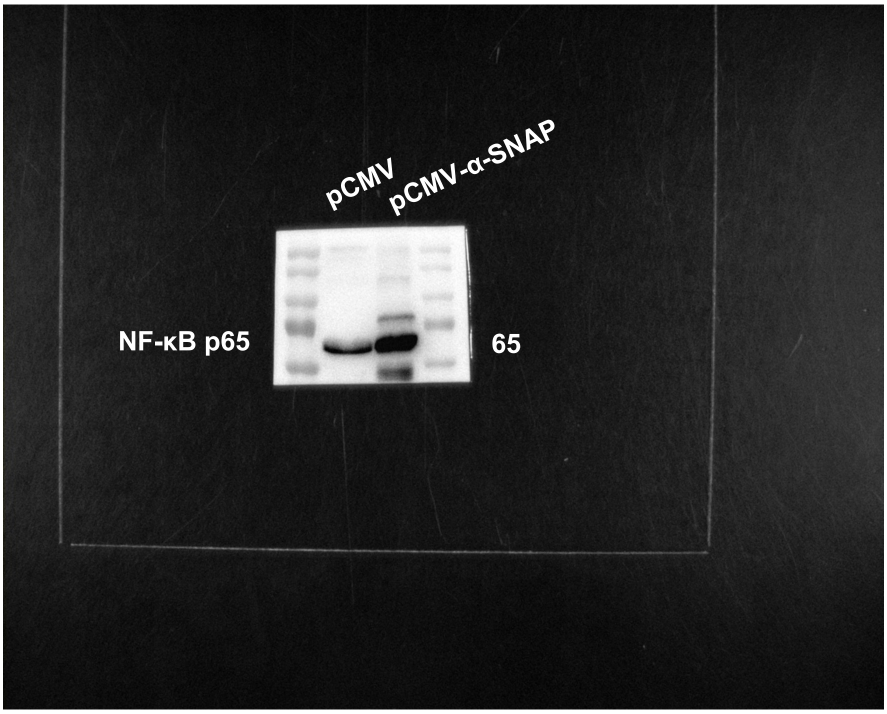

Full unedited gel for Figure 5.I

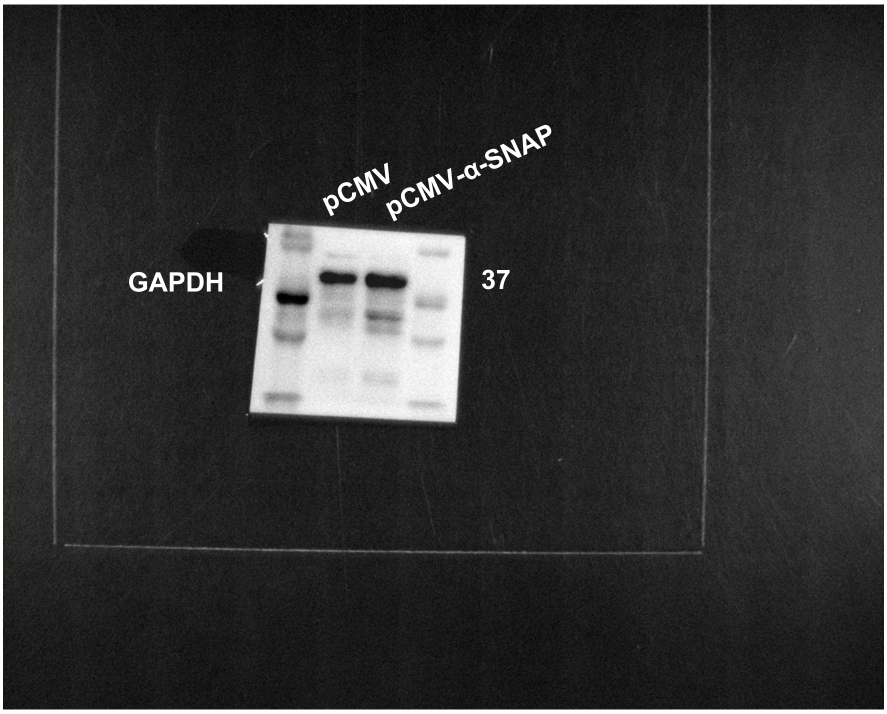

Full unedited gel for Figure 5.I

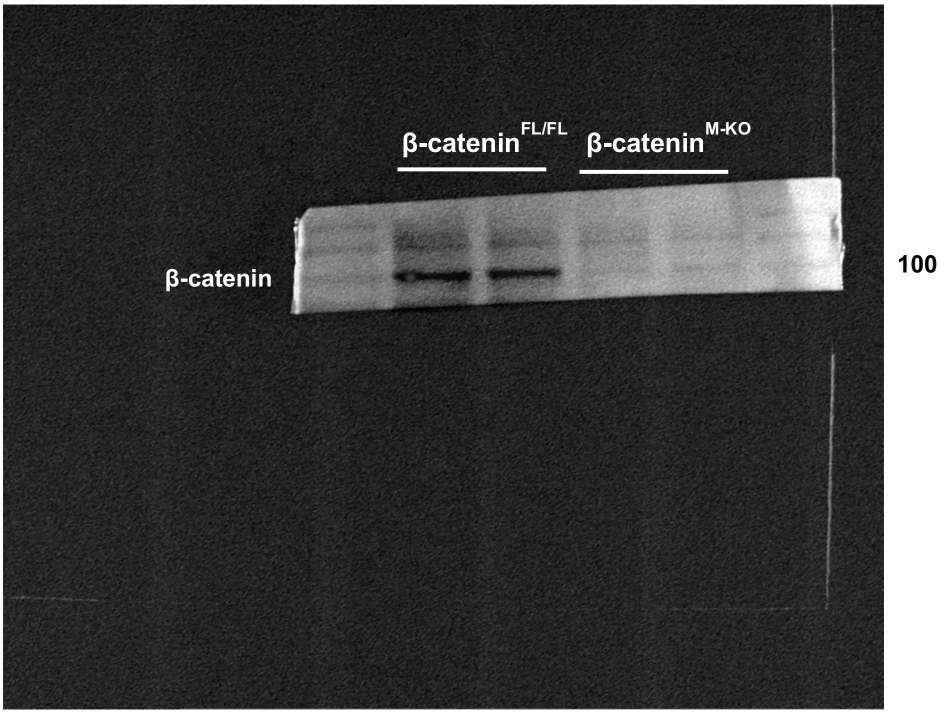

Full unedited gel for Supplemantary figure 2. B

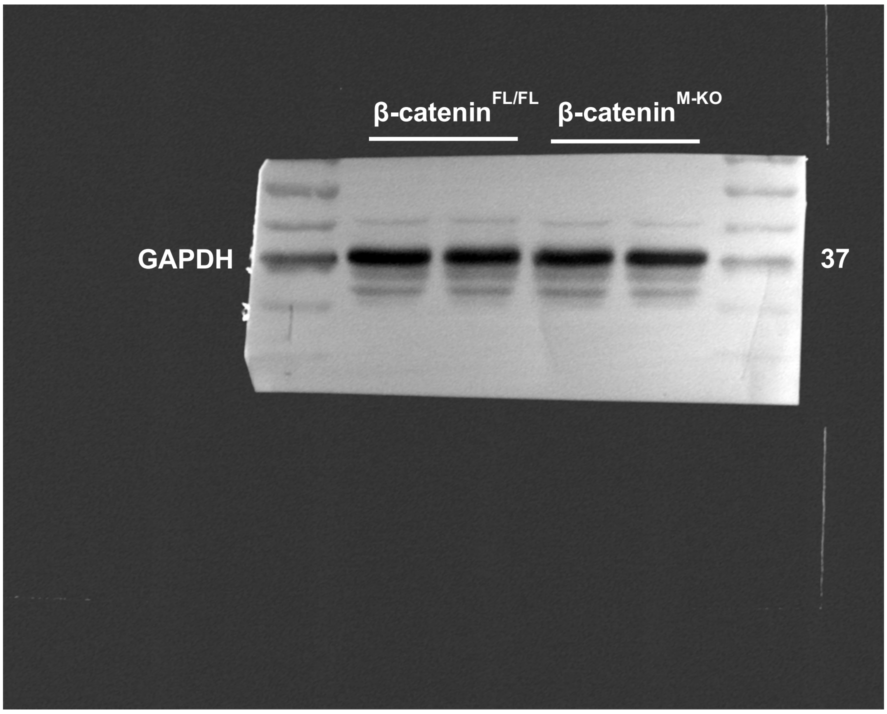

Full unedited gel for Supplemantary figure 2. B

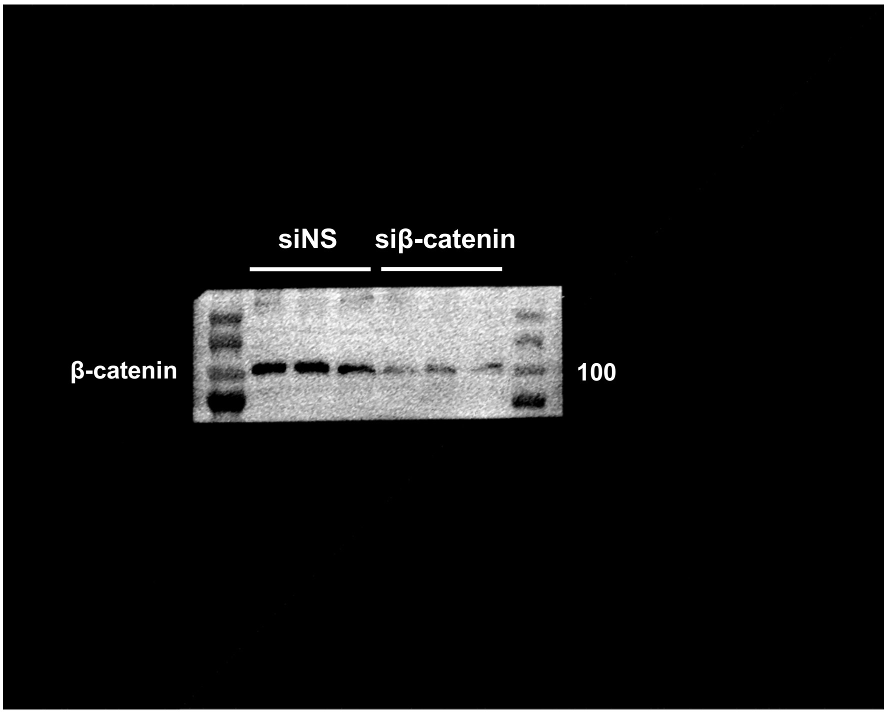

Full unedited gel for Supplemantary figure 3. A

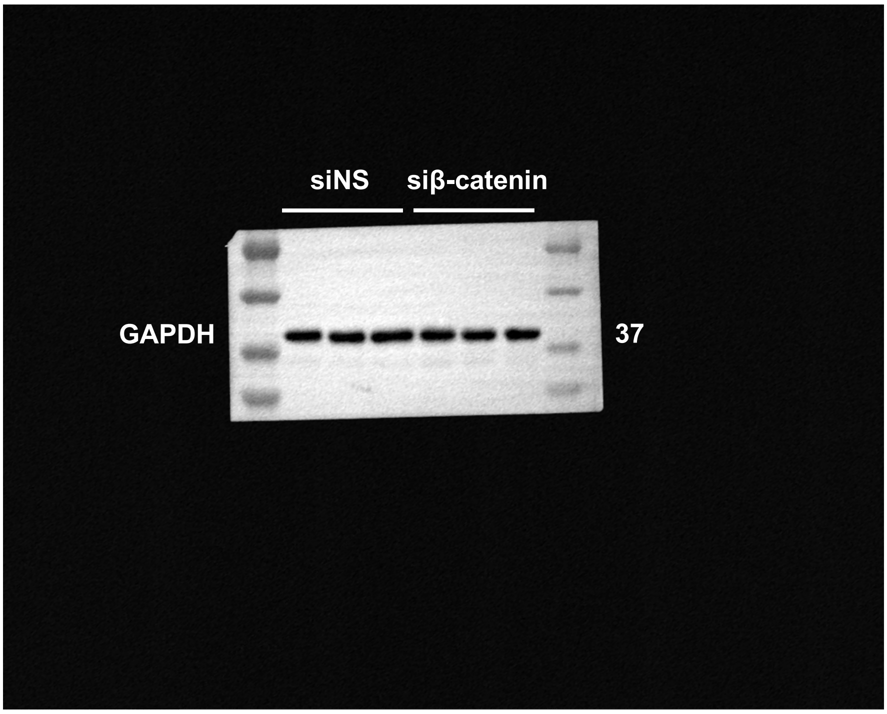

Full unedited gel for Supplemantary figure 3. A

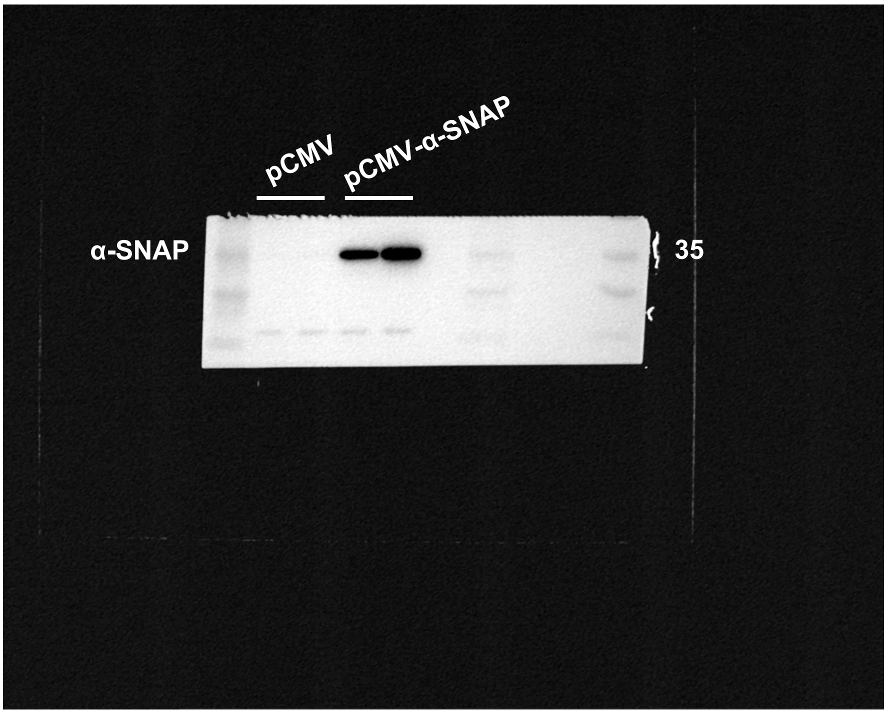

Full unedited gel for Supplemantary figure 3. B

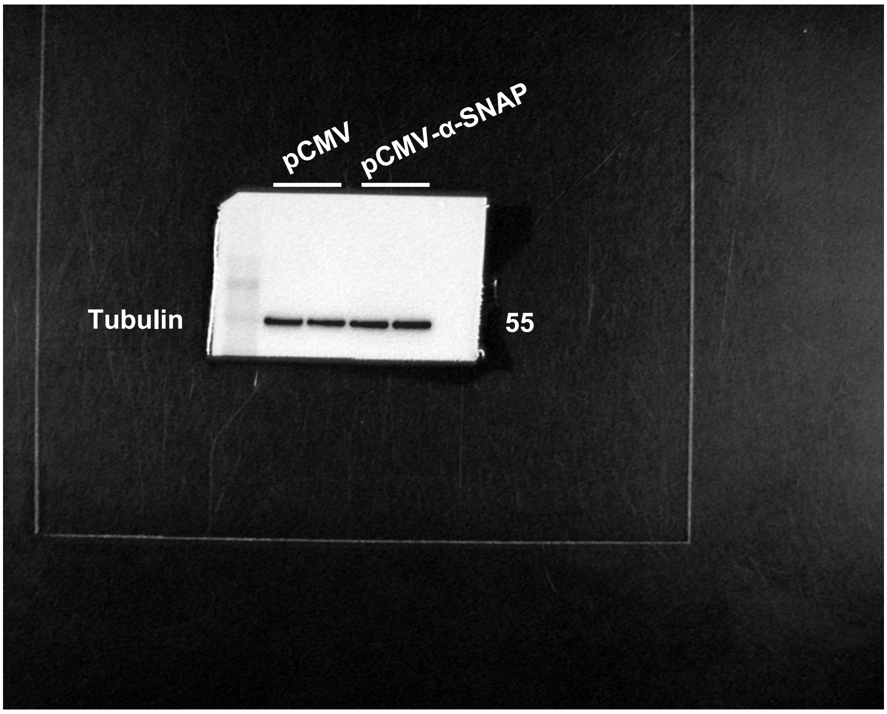

Full unedited gel for Supplemantary figure 3. B

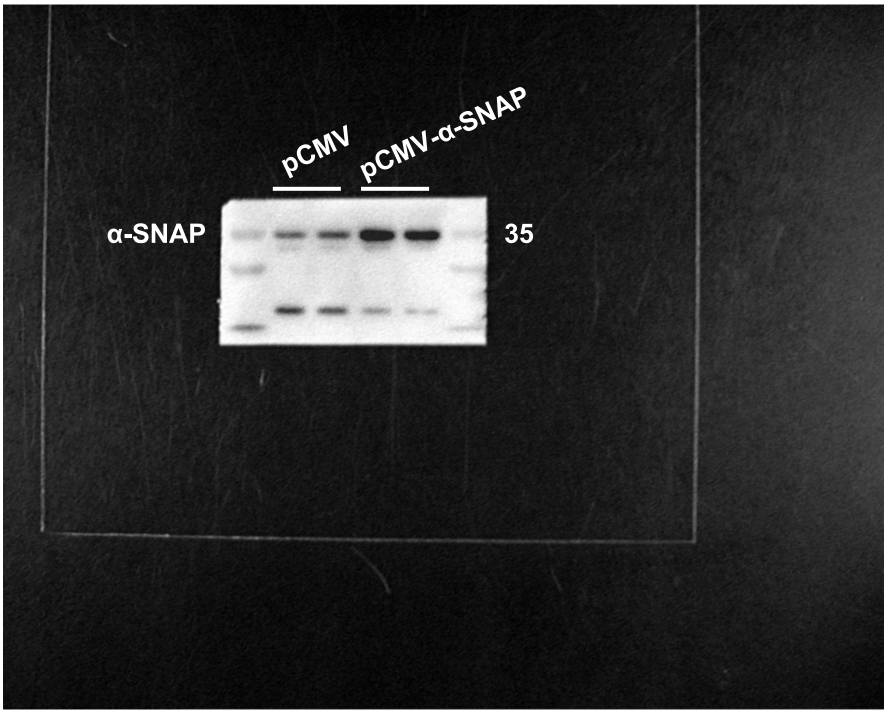

Full unedited gel for Supplemantary figure 3. C

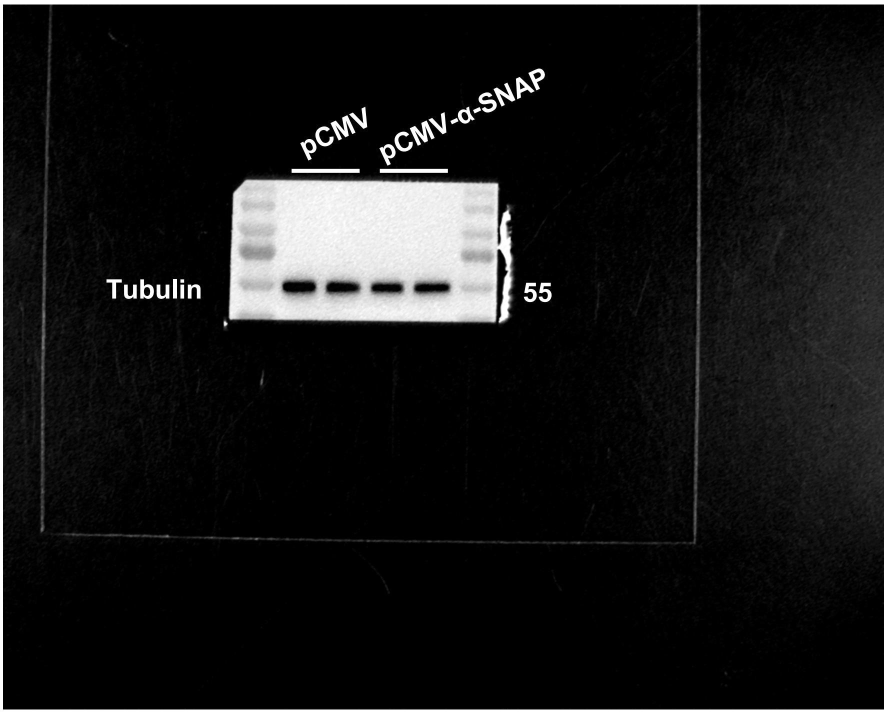

Full unedited gel for Supplemantary figure 3. C
